# Multi-character approach reveals a new mangrove population of the Yellow Warbler complex, *Setophaga petechia*, on Cozumel Island, Mexico

**DOI:** 10.1371/journal.pone.0287425

**Published:** 2023-06-22

**Authors:** Salima Machkour-M’Rabet, Waldemar Santamaría-Rivero, Alexander Dzib-Chay, Leopoldo Torres Cristiani, Barbara MacKinnon-Haskins

**Affiliations:** 1 Laboratorio de Ecología Molecular y Conservación, Departamento de Conservación de la Biodiversidad, El Colegio de la Frontera Sur (ECOSUR), Chetumal, Quintana Roo, México; 2 Transformación Arte y Educación A.C., Delegación Miguel Hidalgo, Ciudad de México, México; 3 Amigos del Delfín S.C. de R.L. de C.V. Celestún, Yucatán, México; 4 Amigos de Sian Ka’an A.C. Cancún, Cancún, Quintana Roo, México; Laboratoire de Biologie du Développement de Villefranche-sur-Mer, FRANCE

## Abstract

The *Setophaga petechia* complex includes 43 subspecies distributed within the new world, of which some are migratory and others are resident, with only two resident subspecies in the Mexican Caribbean: *Setophaga petechia bryanti* a mangrove subspecies belonging to the *erithachorides* group resident on the mainland of the Yucatan Peninsula and *Setophaga petechia rufivertex* endemic to Cozumel Island and belonging to the *petechia* group. Recently, a new population of individuals presenting intermediate phenotypic traits and living in mangrove ecosystems was discovered and reported for Cozumel Island. In this study, we used a multi-character approach including genetic (five ISSR genetic markers), morphometric (eight traits), phenotypic (four characteristics of males), and acoustic dataset (11 parameters) to understand the process of differentiation and the status of these new island individuals in relation to the two well-established subspecies using a total of 60 individuals (20 for each group). Through multivariate analyses based on different dataset used in our study, we show how the new population is related to the endemic island subspecies, *S*. *p*. *rufivertex* and to the mainland subspecies, *S*. *p*. *bryanti* while demonstrating finite differences. We conclude that the new population of *S*. *petechia* on Cozumel Island is a well-established population with high level of differentiation.

## Introduction

Oceanic islands are considered a hotspot of biodiversity [1] and highly vulnerable to the decline in their populations particularly for bird species [2]. Due to their geographic isolation that favors processes such as founder events, selection, and genetic drift among others that lead to phenotypic and genetic differentiation, island species represent a good opportunity to study diversification processes [3, 4]. Birds are widely used as models to study diversification processes [e.g., 5–9]. To understand the origin of these variations, different kinds of information can be used [6]. For example: morphology, particularly bill characteristics [8, 10, 11], acoustic [12, 13], genetic [14, 15], phenotypic [16, 17], and even more accurately the multi-character approaches [e.g., 5, 6, 9]. One mechanism for diversification that can lead to population differentiation and ultimately to speciation, is hybridization [references in 18]. Some studies have documented species diversification through hybrid processes [10, 18–20] showing the importance of hybrid zones in bird evolution.

Yellow Warbler is a complex of 43 subspecies based on plumage color and pattern [21, 22]. This complex of subspecies presents the most widespread breeding range of the wood-warblers and the strongest geographic variation [22]. All subspecies are classified into three main groups based primarily on plumage (amount of chestnut color on the head of the male) as well as structure (e.g., primary projection) [21, 22]. (1) The Yellow Warbler (*aestiva* group), including nine subspecies that are highly migratory, of continental North America characterized by the general absence of chestnut color on the head except on the crown, where, if present, it is diffuse or concentrated on the feather shafts, and all have longer primary projection in relation to the other two groups. (2) The Golden Warbler (*petechia* group) including 18 subspecies that are resident on the Caribbean islands (with extreme south Florida) with generally a well-defined chestnut crown, except for *S*. *p*. *ruficapilla* from Martinique. (3) The Mangrove Warbler (*erithachorides* group) with 16 resident subspecies with a chestnut head that are distributed along the mangrove covered coasts of Mexico, Middle America, and northern South America. Initially, each of the three groups of *Setophaga petechia* was considered a separate species until 1935 at which point different authors combined these groups in different ways [see details in 21, 22]. The International Ornithological Committee (IOC) list [23] considers the *Setophaga petechia* complex as being composed by a migratory species (*aestiva* group) in the north and a resident species (*erithachorides* and *petechia* groups) in the tropics. The taxonomic relationship of *S*. *petechia* clearly needs to be clarified and remains controversial at present. Although the *aestiva* group has been largely studied, few studies have been conducted on the two resident groups, *petechia* and *erithachorides* [24].

Two subspecies are present in the Mexican Caribbean: (1) *Setophaga petechia bryanti* Ridgway, 1873, distributed in mangrove bordering the Yucatan Peninsula and belonging to the *erithachorides* group (Mangrove Warbler), and (2) *Setophaga petechia rufivertex* Ridgway, 1885, endemic to Cozumel Island and belonging to the *petechia* group (Golden Warbler). The first documented report of a Mangrove Warbler on Cozumel Island presenting phenotypes that combine characteristics of the two subspecies (*S*. *p*. *bryanti* and *S*. *p*. *rufivertex*), where Mangrove Warblers were not known to exist, was made in 2014 [25]. Previously, a mangrove-type warbler was reported in 2008 based on the completely chestnut-colored head by someone who had never seen one before, providing an earlier date for its existence on the island [25]; however, we cannot exclude the possibility of its presence on the island before this date. Subsequent expeditions on the island by the authors, along with assistance from local birdwatchers, provided documentation on a minimum of 40 individuals, while not finding a local population of *S*. *p*. *bryanti*. MacKinnon-Haskins and Dzib-Chay [25] suggested that frequent hurricanes in the region may have moved one or more birds from the mainland to the island as demonstrated previously for the Cozumel Island bird community [26]. However, one or more may easily have crossed over at any time the same as the Yellow-lored Parrot and White-crowned Pigeon are known to do [27]. It should also be noted that a Mangrove Yellow Warbler was found in Arizona, 550 Km from the nearest resident population in Sonora [28], in addition to several documented in the area of San Diego and the Imperial Valley of California, far from potential resident populations in Sonora and Baja California Sur [29]. It is important to highlight that the resident subspecies of the Golden Warbler (*S*. *p*. *rufivertex*) occupies scrubby woodland and mangrove edge [27] and although it is abundant on the island, it has never been found in mangrove ecosystems, habitat of the Mangrove Warbler (*S*. *p*. *bryanti*).

**Fig 1:**
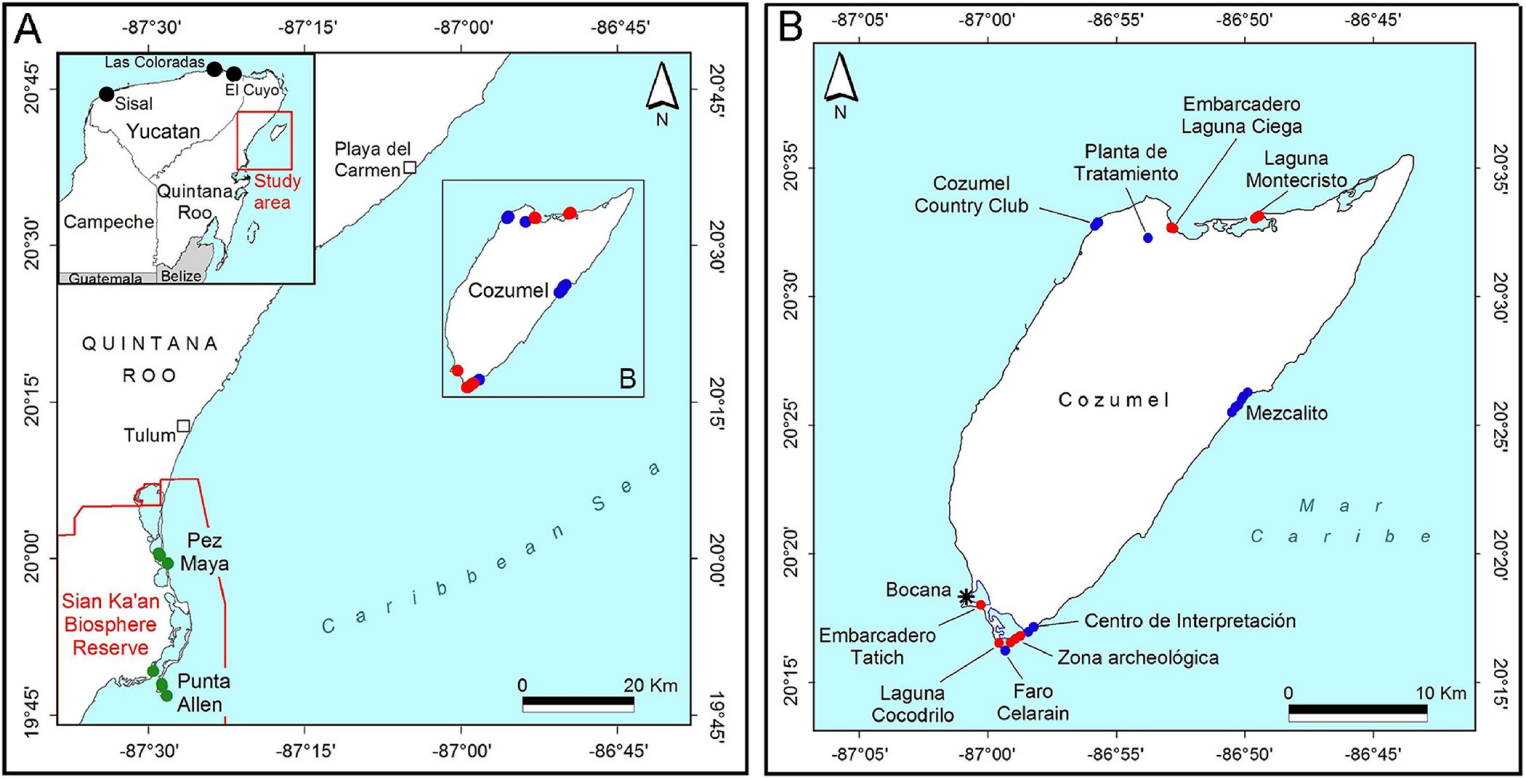
Map of localities in the Yucatan Peninsula, Mexico, where *Setophaga petechia* individuals of three different populations were captured and songs were taped. (A) general view of the area, (B) detail of individual collections on Cozumel Island, (green circle) *Setophaga petechia bryanti*, (blue circle) *Setophaga petechia rufivertex*, (red circle) new island mangrove population, and (black circle) collection points for song data. Source for maps: Map A from *INEGI (2019)*, *state political division*, *scale 1*:*250000*”, and Map B from: Layer downloaded for free from *http*:*//www*.*efrainmaps*.*es*. *Carlos Efraín Porto Tapiquén*. *Geografía*, *SIG y Cartografía Digital*. *Valencia*, *España*, *2020*.

To date, very few genetic studies have been realized on the *Setophaga petechia* species, and the majority have focused on phylogeographic relationships within or between groups, principally the *aestiva* (migratory) group. The first phylogenetic study of *S*. *petechia* [30] showed that the migratory group (*aestiva*) is different from the tropical sedentary group (*erithachorides*). Milot et al. [31] documented the presence of two genetic clades of *S*. *petechia* for Canada and Alaska with a regional genetic subdivision. A more recent genetic study [32] was interested in understanding the evolutionary history of Yellow Warbler with a focus on the *aestiva* group, using 124 northern Yellow Warbler (*aestiva* group), three Mangrove Warbler (*erithachorides* group), and four Golden Warbler (*petechia* group) in their phylogenetic analysis [see Fig 1 in 32]. They showed that resident subspecies (*petechia* and *erithachorides*) formed a separate clade together with the southern lineage of the *aestiva* group. At the same time, Boulet et al. [33] studied the migratory pattern of the northern Yellow Warbler (*aestiva*) using a multi-character approach which include mtDNA analysis. The more recent study [3] focused on *Setophaga petechia aureola* Gould, 1839, the subspecies living in the Galapagos and Cocos Islands. They highlighted the origin of this subspecies and demonstrated the presence of four clusters among all islands [see Fig 3 in 3]. We conducted, for the first time, a study of the Mexican Caribbean resident populations of *S*. *petechia* including genetics.

Plumage color and pattern are the key generally used to distinguish subspecies of *S*. *petechia*. Nevertheless, some subspecies may not respond to this rule [e.g., 34]. Different studies show some relation between plumage coloration and mating success [e.g., 35–37]. When we consider morphological characteristics of the *S*. *petechia* complex, studies principally highlight differences among groups for wings [e.g., 38] or bill morphology [e.g., 39], and for tarsus length [e.g., 39]. Some studies point to the differences between the song of *aestiva* group and the Mangrove Warbler [40].

Considering the aforementioned information, our study used a multi-character approach based on genetic data combined with the more commonly used morphometric, acoustic, and phenotypic information to evaluate the relationship between *S*. *p*. *bryanti* a Mangrove Warbler (*erithachorides* group), *S*. *p*. *rufivertex* a Golden Warbler endemic on Cozumel Island (*petechia* group), and a new population of Yellow Warbler on Cozumel Island inhabiting the mangrove ecosystem.

## Materials and methods

### Ethics statements

The manipulation of birds was authorized by the Secretary of the Environment and Natural Resources of Mexico (SEMARNAT for its acronym in Spanish) under permit number SGPA/DGVS/000649/18. All birds were gently handled when taking morphological data, genetic sampling, and photographs, after which they were released in the same place in which they were captured. Field work was carried out during the non-reproductive season.

### Morphological, phenotypic, and genetic approaches

#### Study area

The study area is located in the state of Quintana Roo, Mexico (Fig 1). Individuals of *S*. *p*. *rufivertex* and the new island mangrove population were captured in locations within the Cozumel Island Flora and Fauna Protected Area (20°32’43.85”N-86°52’51.91”O) and the Laguna Colombia State Ecological Reserve (20°28’59.17”N-86°97’02.33”O) on Cozumel. The climate on the island is humid and warm, with a annual rainfall of 1570 mm and a mean annual temperature of 25.5°C [41]. Individuals of *S*. *p*. *bryanti* were collected at two locations within the Sian Ka’an Biosphere Reserve (20°00’86.50”N-87°48’23.67”O) on the central mainland coast of Quintana Roo. The climate in this area is sub-humid and warm with a annual rainfall of 1128 mm and a mean annual temperature of 26.5°C [41].

#### Sample collection and data acquisition

For each subspecies and the new island mangrove population, we caught 20 individuals using mist-nets (12 x 2.6 meters) between August 7 and October 15, 2018, obtaining a total of 60 individuals. On Cozumel Island, warblers were collected in the coastal dune vegetation, mangrove, and secondary forest while in the Sian Ka’an Biosphere Reserve the warblers were captured in the coastal mangrove. In order to help in the capture of birds, a song playback of each subspecies was used for a maximum of two minutes [42]. A unique colored plastic band was placed on the tarsus of each individual captured to avoid sample duplication and to allow for a long-term-follow-up on individuals. Age and sex of each bird was evaluated based primarily on cranial ossification, plumage, and bill color [22, 27, 38].

**Fig 2:**
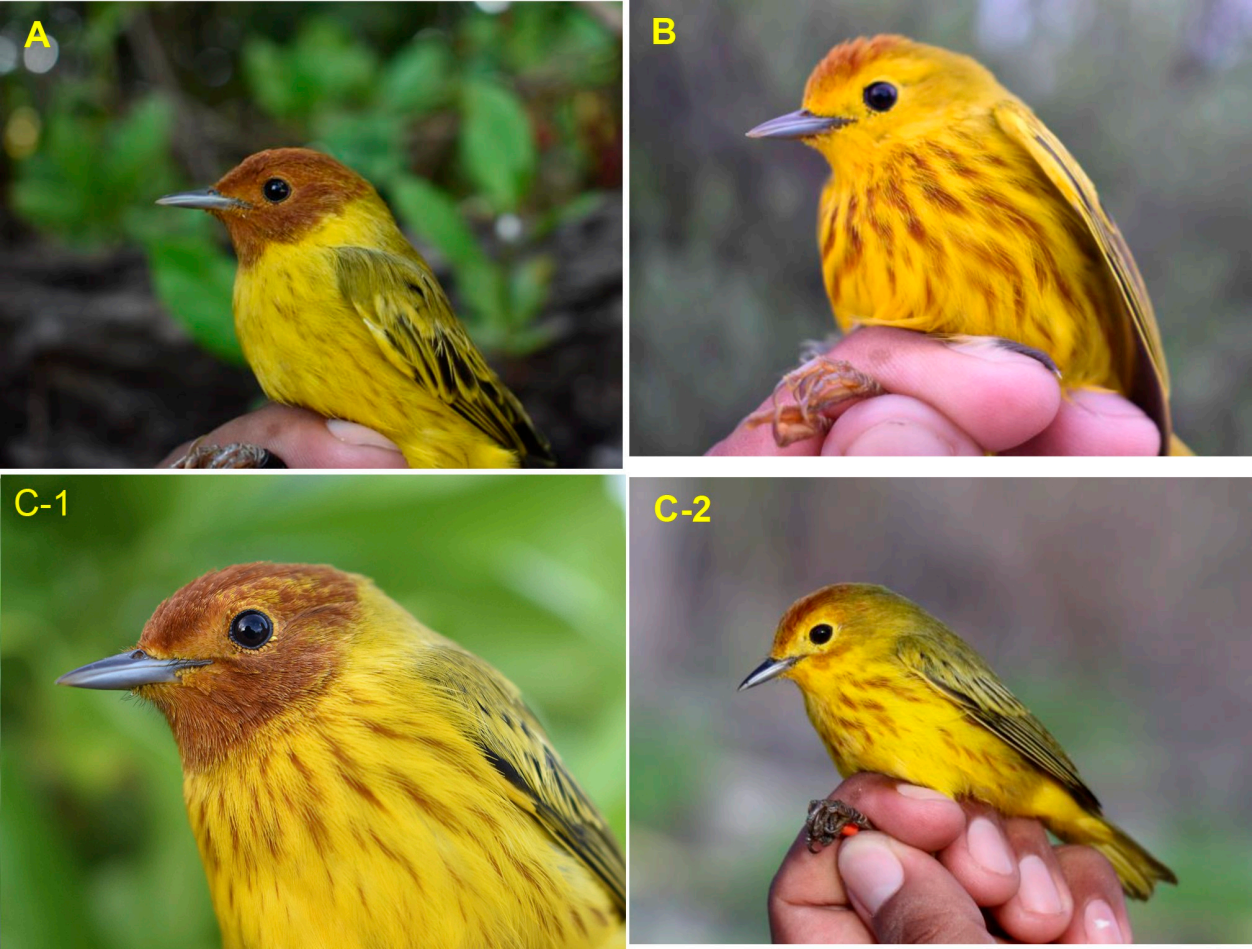
Variation of head coloration and streaked breast of adult male *Setophaga petechia* encountered in the Yucatan Peninsula, Mexico. (A) Mangrove Warbler, *S*. *p*. *bryanti*, with its characteristic complete chestnut-colored head and thinly streaked breast, found in mangrove on the mainland coast, (B) Golden Warbler, *S*. *p*. *rufivertex*, with only chestnut-colored crown, heavily streaked breast, yellow lores and faint throat streaks, found in deciduous forest and dune scrub on Cozumel Island, and (C) the island mangrove population, with two examples of coloration of head: C-1 almost complete chestnut colored head, yellow lores and broadly streaked breast, and C-2 with partially chestnut-colored head and throat, yellow lores and broadly streaked breast. Photo credit: Waldemar Santamaria-Rivero.

A total of eight morphometric traits were measured for each individual: wing length, tarsus length, tail length, bill length, bill width, bill height, total length, and weight; photos were taken of each at different angles. Four phenotypic characteristics of adult male individuals were considered and defined as follows: (1) amplitude and evenly chestnut-colored head (typical of *S*. *p*. *bryanti*), only chestnut coloration of the crown (typical of *S*. *p*. *rufivertex*), and intermediate coloration of head with yellow lores (details of coloration are fully explained in Fig 2 and S1 Fig), (2) width of the chest streaks considering broad or narrow, (3) presence or absence of colored streaking on the back, and (4) presence or absence of streaking on the undertail coverts.

For genetic analysis, a blood sample (around 100 μl) was taken by jugular venipuncture [43], after which one part of the blood was conserved on a small paper filter (1 cm^2^) and another part was kept in a Buffer Queen’s Lysis solution [44]. All blood samples were transferred to the Laboratory of Molecular Ecology and Conservation at the El Colegio de la Frontera Sur research center (Quintana Roo, Mexico). Upon completing these procedures, the birds were released in the same location where they were captured.

#### Body and bill size analyses

All morphological measurements were considered for analysis except weight which fluctuates between individuals and time of day [5]. Only adult individuals were considered for morphological analysis.

To determine the relation between the two subspecies and the new island mangrove population, we performed a linear discriminant analysis (LDA). Additionally, we processed a non-parametric MANOVA (NPMANOVA also known as PERMANOVA) based on Euclidean distances using 9,999 permutations followed by a pairwise post-hoc comparison test (normality of variables was tested using the Shapiro-Wilk test). To evaluate the contribution of morphological traits for the difference detected between the two subspecies and the new island mangrove population, we performed a Similarity Percentage (SIMPER) procedure considering Euclidean distances as dissimilarity measure. Finally, a Kruskal-Wallis test followed by a Dunn’s post-hoc test was applied to each of the morphological traits considering the three populations. All morphological analyses were processed using the free software PAST 3.21 [45].

#### Phenotypic characterization

To evaluate the relationship between the three populations based on phenotypic information, data was converted into a binary matrix and a correspondence analysis (CA) was applied using the software PAST 3.21. For analysis, the evaluation of the age of individuals was considered as SY for individuals in its second calendar year and ASY for individuals in at least its third calendar year [46].

#### Genetic laboratory procedures and analyses

*Laboratory procedures*. DNA was extracted using the salt protocol [47] maintaining samples at temperature of less than 5°C. DNA concentration was determined by fluorometry (Qubit 2.0, INVITROGEN) and DNA quality was assessed by electrophoresis in agarose gel (TAE 1%) using GelRed (BIOTUM) as a post-staining method.

A total of 14 ISSR genetic markers were screened to identify the best genetic markers in term of polymorphism and unambiguous scoring. From these genetic markers, four did not produce results, four gave a very low level of polymorphism, and from others, five were selected to conduct our study: (AC)_8_C, (AG)_8_C, BDB(ACA)_5_, (GAG)_5_GC, (GGTA)_4_ (S1 Table). PCR amplifications were carried out in a 15 μL reaction volume containing ~20 ng of template DNA (2 μL), 1.5 μL of 5X Green Buffer (PROMEGA), 200 μM of each dNTP (0.3 μL) (dNTP mix, PROMEGA), 3 mM of MgCl_2_ (1.8 μL) (PROMEGA), 1 μM of primer (0.3 μL) (INTEGRATED DNA TECHNOLOGIES), 1.25 U of Taq Polymerase (0.25 μL) (GoTaq Flexi, PROMEGA), adjusting the final volume with ultrapure water. All amplifications were processed in a gradient thermocycler (T100 thermal cycler, BIO-RAD) under the following cycling conditions: initial denaturation at 94°C for 4 min, 39 cycles of denaturation at 94°C for 45 s, primer annealing temperature 52.5–63°C, depending on the ISSR primer (S1 Table), for 45 s, an extension at 72°C for 90 s, and a final extension at 72°C for 10 min. DNA banding patterns were visualized by electrophoresis on a 2% agarose gel with a 1X TBE buffer and post-staining with GelRed (BIOTIUM), at 110 V for 2 h. Lengths of amplification products were estimated using a 100 bp DNA ladder (PROMEGA). DNA fragments were visualized and digitized using an imaging system (Photodoc-IT 65, UVP). ISSR reproducibility in our experiments was tested considering the following criteria: (1) 10% of samples from the total sample size [48] was used, (2) the same DNA extract was used in the reproducibility test and experiment, and (3) each DNA sample was replicated three times; then, we determined the genotyping error rate [48, 49]. The mean error rate per locus was 2.37% which is the average reported in other studies [49] indicating that our data was suitable for analysis.

The patterns of ISSR bands were scored as diallelic characters (presence scored as 1, absence scored as 0) from which a binary database was elaborated for subsequent analysis. Only bands that could be scored consistently with clear and unambiguous patterns were kept for analysis.

*Analyses*. Genetic diversity of the two subspecies and the new island mangrove population of the *Setophaga petechia* complex was determined using the percentage of polymorphism loci (*PPL*) and the expected heterozygosity (*H*_*e*_) using GENALEX 6.5 [50, 51] and POPGENE 1.31 [52]. Furthermore, global heterozygosity was presented considering the *H*_T_ (total heterozygosity if all three populations are considered as one) and *H*_S_ (mean heterozygosity within each of the three populations) parameters determined by POPGENE 1.31. Additionally, the program HICKORY v1.1 [53] was used to determine genetic diversity (*h*_*s*_) based on the Bayesian method that does not consider prior assumption of Hardy-Weinberg equilibrium that is recommended for dominant markers. The three principal models proposed by HICKORY were run with the default parameters: 1) the *full model* which considers population differentiation (*θ* analogous to *F*_*ST*_) and inbreeding (*f* analogous to *F*_*IS*_), 2) the *θ* = 0 model which considers no population differentiation, and 3) the *f* = 0 model which considers no inbreeding. The choice of model is based on the value of DIC (Deviance Information Criterion) and Dbar parameters, lower are better [54, 55]. Finally, we applied a nonparametric Kruskal-Wallis test (Shapiro-Wilk and Anderson-Darling tests showed the non-normality of data) considering the *H*_*e*_ value at each loci as variable to test the difference in genetic diversity among the three populations of the complex. Tests were made with the software PAST 3.21.

Genetic differentiation was determined using the *Φ*_*PT*_ values, an analogue of Wright’s *F*_*ST*_, determined for all pairs of the three populations (9,999 permutations in GENALEX 6.5), and with the *θ*^*II*^ value, the most relevant statistic to evaluate the genetic differentiation among contemporaneous populations proposed by HICKORY v1.1. To test if the new island mangrove population is closer to the mainland subspecies (*S*. *p*. *bryanti*) or to the island subspecies (*S*. *p*. *rufivertex*), two different scenarios were tested through a hierarchical analysis of molecular variance (AMOVA; 9,999 permutations) using GENALEX 6.5. Furthermore, to reinforce the results suggested by *Φ*_*PT*_ and hierarchical AMOVA, genetic structure among the three populations was evaluated through different methods: (1) the Bayesian model-based clustering implemented in the program STRUCTURE [56, 57] was performed to identify the most probable number of clusters (*K*). The admixture and allele correlated frequency models were performed. The program was run 10 times for different values of *K* (from 1 to 6) with the Markov Chain Monte Carlo algorithm using a burn-in period of 100,000 steps followed by 100,000 steps. To identify the most probable number of *K*, we determined the maximal value of Ln *P(D)*, the value that indicates the start of "more-or-less plateaus" for larger *K* [56], and additionally, we determined the Δ*K* [58] that can be found in the STRUCTURE HARVESTER website [59], (2) the principal coordinate analysis (PC_O_A), an exploratory analysis without a priori assignment of individuals was processed using GENALEX 6.5, and finally (3) a distance-based method was applied through a Neighbour-Joining tree using the SPLITS TREE4 [60].

### Acoustic study

#### Study area and data acquisition

During the song recording, birds were approached very carefully using a play back of male individuals and done during the non-reproductive season to avoid any perturbation. Songs of *S*. *p*. *bryanti* were recorded at the following two locations on the coast of Yucatan: between Las Coloradas and El Cuyo (21°33’05”N-87°49’35”O), and near Sisal (21°05’49”N-90°11’31”O) (Fig 1A). At both sites, songs were taped in mangrove areas away from cities to avoid noise contamination. For *S*. *p*. *rufivertex* and the new mangrove population on Cozumel Island, areas were selected where each one was known to reside without the presence of the other. For *S*. *p*. *rufivertex*, dirt paths with typical vegetation of secondary forest were selected, whereas for the new mangrove population coastal mangrove habitats were visited. Songs were collected between 2014 and 2019 between the hours of 6:00 am and 10:00 am and in the afternoon between 3:00 pm and 5:00 pm. A total of 240 recordings were made for the three populations exclusively of males, from which 60 were selected (20 per population). Male songs were recorded at close range (3–6 m) using a TASCAM DR-60DmkII 4-Channel Portable recorder and a SENNHEISER MKE600 Shotgun microphone.

#### Analyses

Bird songs were analyzed using SOUND FORGE 11 PRO (SONY) with a Fast Fourier Transformation (FFT length 512, FFT overlap 93, sampling frequency 22,050 Hz, frequency resolution 43 Hz, sonogram resolution 8,000 and Hanning Window Function). A total of 11 acoustic parameters were determined to describe the song and were classified as follows [61]: (1) acoustic parameters to evaluate differences at syllable level with DurSyl (first syllable duration), FminSyl (minimum frequency of the first syllable), FmaxSyl (maximum frequency of the first syllable), DurSylTr (trill syllable duration), and FdomSylTr (dominant frequency of the trill syllable), (2) acoustic parameters to evaluate differences at phrase level with DurPhr (song phrase duration), Fmin and Fmax (minimum and maximum frequency of the song phrase), and Fdom (dominant frequency of the song phrase), and finally (3) parameters to evaluate song complexity and continuity with OrSyl (number of original syllables per phrase) and TotSyl (total number of syllables per phrase).

Linear discriminant analyses (LDA) were performed to describe the relation between the three populations considering (1) the 11 acoustic parameters, (2) the five acoustic parameters characteristic of syllable level, and (3) the four parameters characteristic of phrase level. Furthermore, non-parametric MANOVA (NPMANOVA also known as PERMANOVA; normality of variables was tested using the Shapiro-Wilk test) based on Euclidean distances (9,999 permutations) followed by a pairwise post-hoc comparison test performed for each situation and for the two acoustic parameters of song complexity. Finally, a non-parametric Kruskal-Wallis test followed by a Dunn’s post-hoc test was applied to each of the acoustic parameters considering the three populations. All song analyses were processed using the free software PAST 3.21.

## Results

### Body and bill size

**Fig 3:**
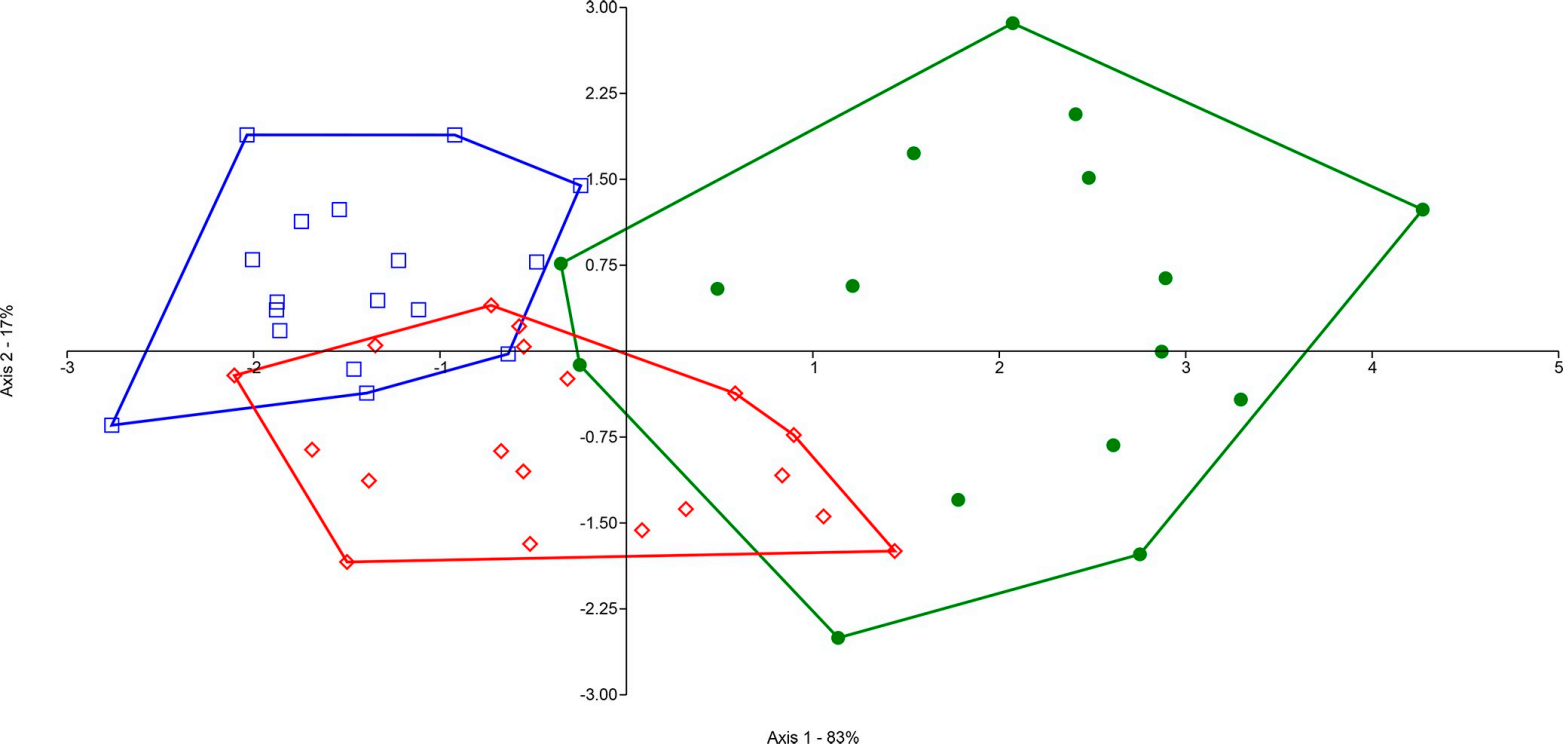
Linear discriminant analysis (LDA) based on seven morphometric traits for the Yellow Warbler complex, *Setophaga petechia* in the Yucatan Peninsula, Mexico. *Setophaga petechia bryanti* from mainland (green color), *S*. *p*. *rufivertex* (blue color) and the new mangrove population (red color) from the Island.

**Fig 4:**
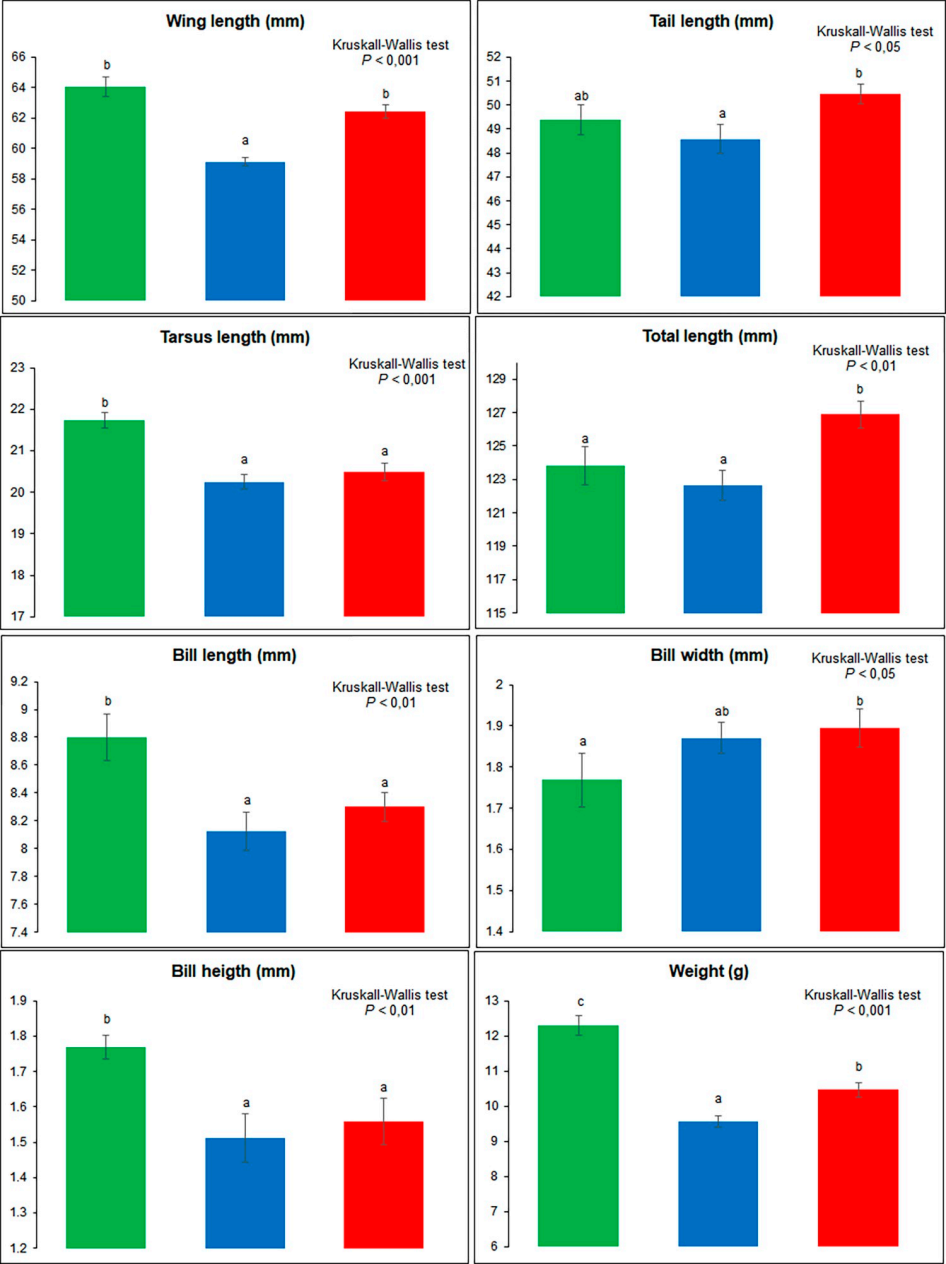
Histograms for morphological traits of the *Setophaga petechia* complex in the Yucatan Peninsula, Mexico. *Setophaga petechia bryanti* (green), *S*. *p*. *rufivertex* (blue), and the new mangrove population (red). Mean and standard error are presented. Letters above each box represent intergroup differences followed by the Dunn post-hoc test.

Since no difference between sexes was observed for any of the morphological traits, all adult individuals were used in our analyses. The LDA (Fig 3) permits separation of the three populations with few individuals overlapping two groups. First discriminant axis showed a very high separation between mainland (positive side; green color in Fig 3) and island (negative side; red and blue colors in Fig 3) populations, while the second discriminant axis separated the two-island populations. The PERMANOVA showed a highly significant difference between the three populations (*F* = 8.79, DF = 2, *P* = 0.0001) as well as for each pair of populations (S2 Table). SIMPER analysis showed medium to high overall average dissimilarity following analyses of groups (from 67.87 to 82; S3 Table). Whatever the association of populations analyzed, the morphological traits that contribute the most (cumulating more than 90%) to differentiate the three populations are total length, wing length, and tail length, while the bill measurements contribute the least to differentiate the three populations (S3 Table). Finally, the statistical analysis of each morphological trait among the three populations (Fig 4) suggests that the new island mangrove population is globally bigger (greater total length and tail length) than the two subspecies, whereas *S*. *p*. *rufivertex* which lives in scrubby woodland and coastal dune vegetation has shorter wings than those living in mangrove vegetation (*S*. *p*. *bryanti* and new island population). Generally, other morphological traits are longer for the mainland mangrove subspecies (*S*. *p*. *bryanti*) than for the two island populations (Fig 4).

### Phenotypic characterization

**Fig 5:**
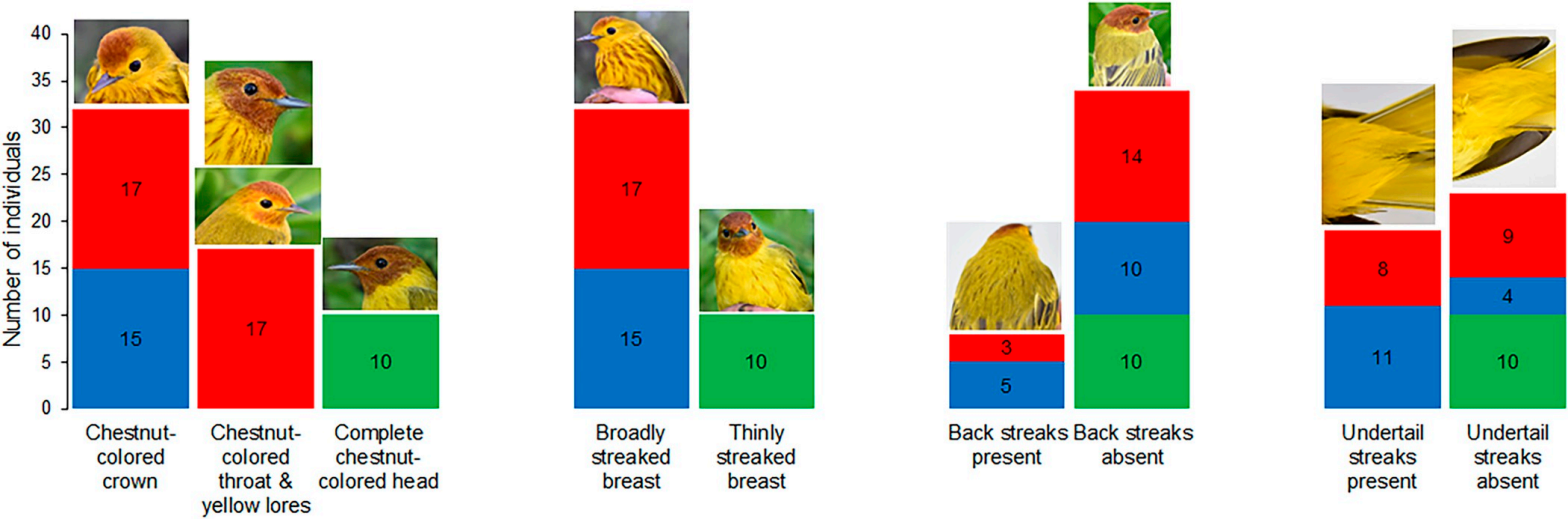
Stacked bar chart of phenotypic characteristics of three populations from the *Setophaga petechia* complex (adult males). *Setophaga petechia bryanti* (green), *S*. *p*. *rufivertex* (blue), and the new island mangrove population (red). The number inside the box represents the number of adult males presenting this characteristic. Photo credit: Waldemar Santamaria-Rivero and Barbara MacKinnon-Haskins.

**Fig 6:**
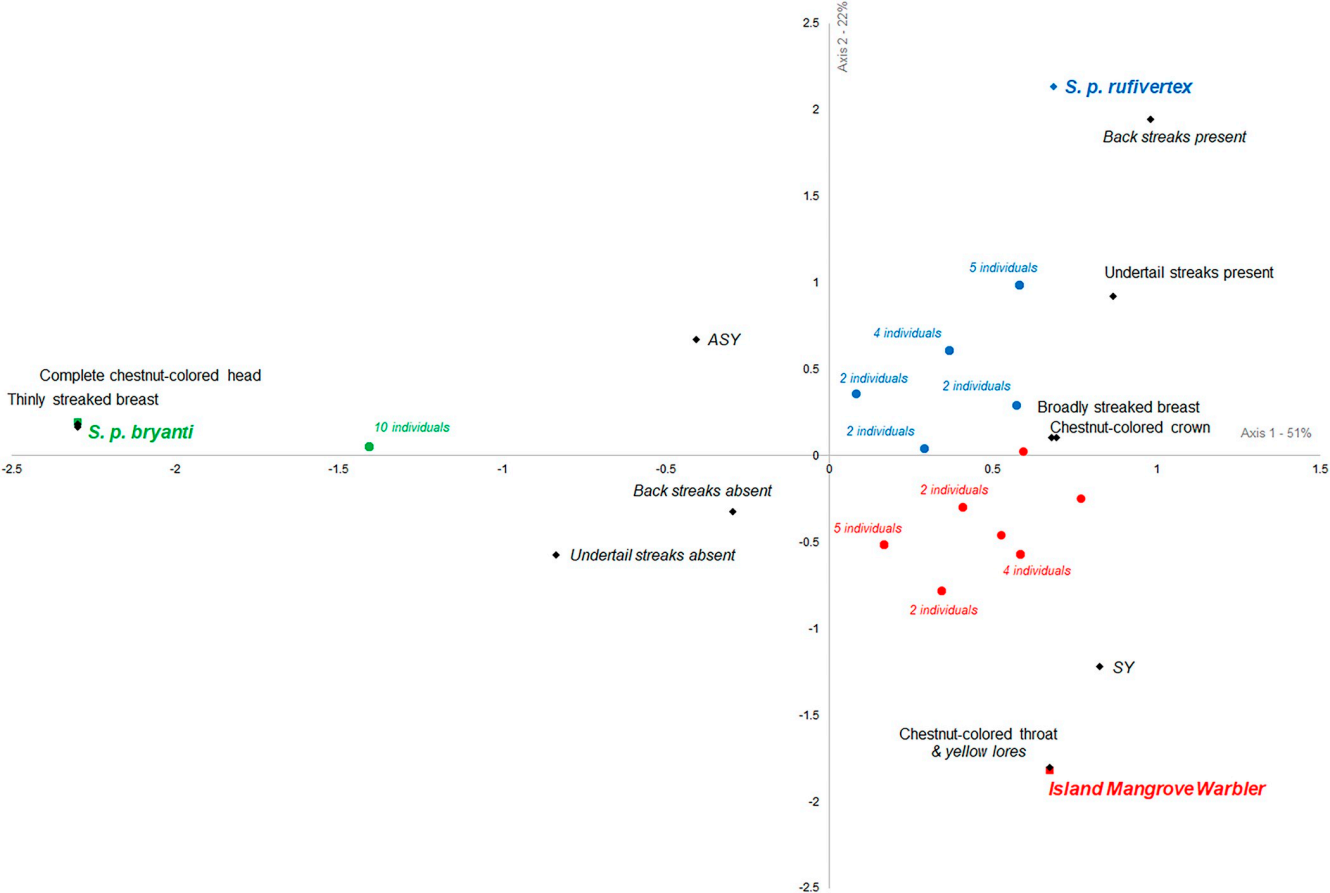
Correspondence analysis of the phenotypic characteristics of the two subspecies and the new island mangrove population from the *Setophaga petechia* complex in the Yucatan Peninsula, Mexico. SY (second calendar year individuals) and ASY (third year or older individuals) follow [46].

Results of phenotypic characterization of males of the two subspecies and the new island mangrove population were resumed in a stacked bar chart (Fig 5) showing the high stability in the phenotypic traits for *S*. *p*. *bryanti* (green in Fig 5) while the new mangrove population (red in Fig 5) showed more variation in some phenotypic characteristics. Correspondence analysis (Fig 6) permitted the separation of the three populations and suggested the same tendency. The subspecies *S*. *p*. *bryanti* (green in Fig 6) showed only one point for all individuals suggesting that all birds are phenotypically very similar, the *S*. *p rufivertex* subspecies (blue in Fig 6) showed five points on the graph for the 15 individuals suggesting some homogeneity in the phenotypic traits, but the new mangrove population showed a wide dispersion of points suggesting more diversity in its phenotypic characterization.

### Genetic

The five ISSR genetic markers used permitted to obtain a total of 57 clear and unambiguous bands (loci) for 60 individuals from the two subspecies and the new island mangrove population. Analysis of data using HICKORY v1.1 showed that the *full model* is best indicated by the lowest values of DIC and Dbar parameters (S4 Table); results presented are those obtained with this model. Considering the three populations, the percentage of polymorphic loci was 84.2% and the mean heterozygosity (*H*_e_ ± SE) was 0.261 (± 0.015). At the subspecies level (Table 1), *S*. *p*. *bryanti* presented lower genetic diversity than the two island populations but it is not significant (*H* = 0.75, DF = 2, *P* = 0.68). Considering the island populations, *S*. *p*. *rufivertex* has slightly higher values than the new population. All values obtained using HICKORY software are slightly higher.

**Table 1:** Genetic diversity parameters determined with GENALEX, POPGENE, and HICKORY v1.1 (*full model*) software for three populations of *Setophaga petechia* complex from Quintana Roo, Mexico.

	n	*PPL*	*H*_e_ ± SE		*h*_s_ ± SD
*S*. *p*. *bryanti*	20	64.9	0.244 ± 0.027		0.306 ± 0.012
*S*. *p*. *rufivertex*	20	77.2	0.277 ± 0.025		0.358 ± 0.011
New population	20	77.2	0.260 ± 0.025		0.338 ± 0.012
*H*_T_ (Mean ± SD)				*H*_S_ (Mean ± SD)	
0.303±0.030 0.389±0.010				0.261±0.024 0.334±0.007	

Number of samples (*n*), percentage of polymorphic loci (*PPL*), expected heterozygosity (*H*_e_ ± SE), estimate genetic diversity evaluated by HICKORY v1.1 (*h*_s_ ± SD), *H*_T_ (total heterozygosity if all three populations are considered as one) and *H*_S_ parameter (mean heterozygosity within each population) for which left values were calculated by POPGENE and right values using HICKORY.

Genetic differentiation between the three populations obtained using both software (GENALEX and HICKORY) gave the same results (respectively *Φ*_*PT*_ = 0.210*** and *θ*^*II*^ = 0.209 also significant (95% credible interval: 0.144–0.277)). The pairwise genetic differentiation showed lower value between both island populations (S5 Table). The best hypothesis to group the populations obtained through the hierarchical AMOVA suggests that both island populations are more closely related (S6 Table).

**Fig 7:**
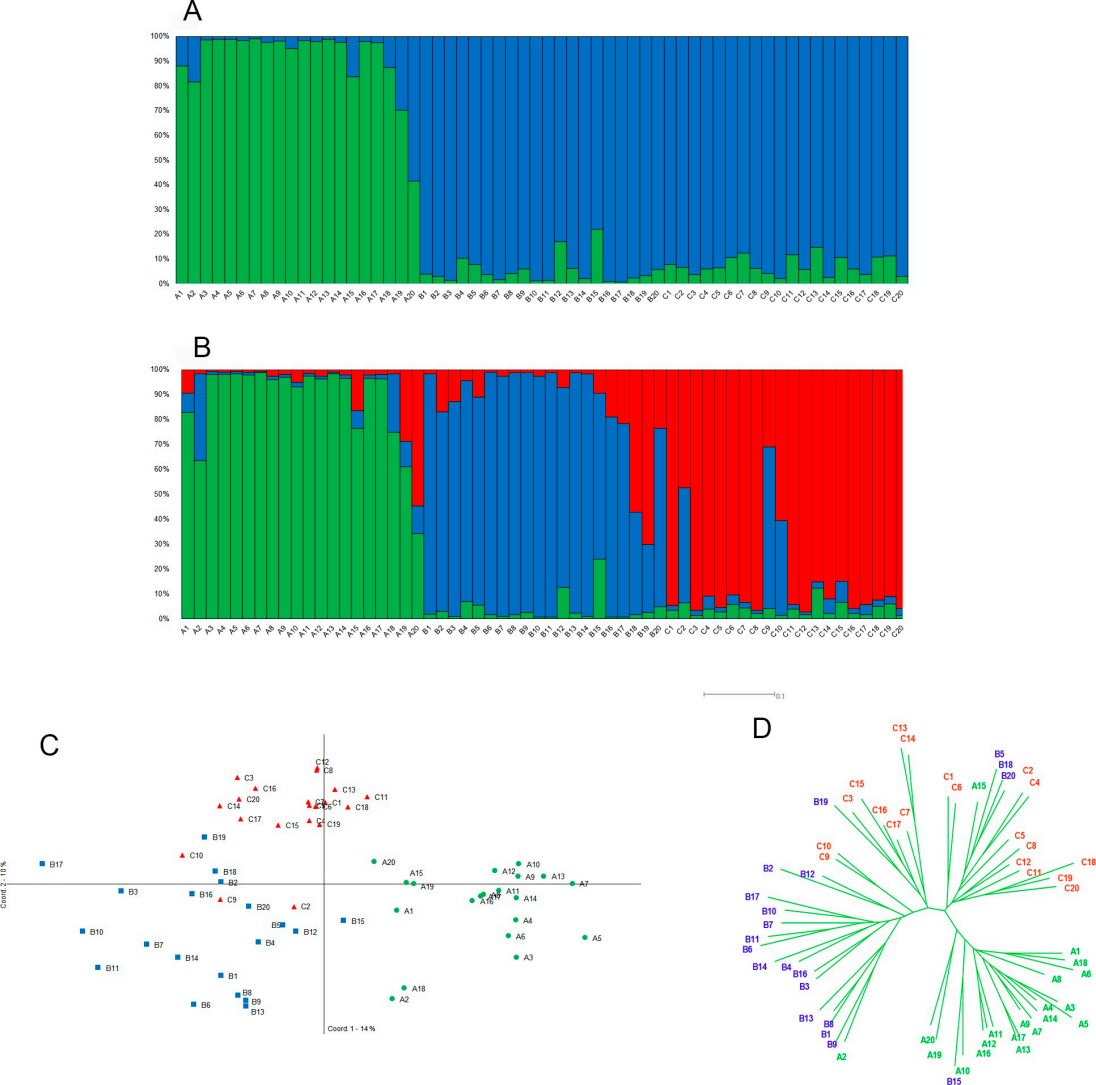
Genetic structure results based on ISSR markers for the two subspecies and the new island mangrove population from the *Setophaga petechia* complex in the Yucatan Peninsula, Mexico. Bayesian analysis computed by STRUCTURE 2.3.1 software where each individual is represented by a single vertical line broken into *K* segments of length proportional to the estimated membership (probability *q*_*i*_) in the *K* clusters: (A) for *K* = 2 as suggested by the Δ*K* method and (B) for *K* = 3 as suggested by *LnP(D)* values. (C) principal coordinate analysis (PC_O_A) processed in GENALEX 6.5, and (D) unrooted Neighbour-Joining tree computed by SPLITS TREE4. *Setophaga petechia bryanti* (green), *S*. *p*. *rufivertex* (blue), and the new island mangrove population (red).

Results of Bayesian analysis suggested that the three populations are grouped into two clusters (S2 Fig) with very high membership probability (> 90%; S7 Table) with the Δ*K* method, whereas the Ln *P(D)* values suggested three clusters (S2 Fig) with a good membership probability for each new cluster (> 80%; S7 Table); consequently, both results are presented. When two clusters are considered, both island populations are grouped (blue lines in Fig 7A) which is in accordance with previous results of genetic differentiation. When three clusters are considered (Fig 7B), each one corresponds to one population (green for *S*. *p*. *bryanti*, blue for *S*. *p*. *rufivertex*, and red for the new island mangrove population) and few individuals present mix genetic profile, principally on the island which is not surprising considering the geographic proximity between both populations in certain areas. Principal component analysis (PC_O_A) also showed a good separation between the three populations (Fig 7C). The X-axis separated the mainland subspecies (*S*. *p*. *bryanti*, green in Fig 7C) from the island populations, whereas the Y-axis permits splitting both island populations (*S*. *p*. *rufivertex* in blue and the new mangrove population in red in Fig 7C). Finally, results obtained from the Neighbor-Joining tree (Fig 7D) confirmed all previous results with an isolation of mainland subspecies (green in Fig 7D) from the two-island populations which split into two clusters (blue and red in Fig 7D).

#### Acoustic

**Fig 8:**
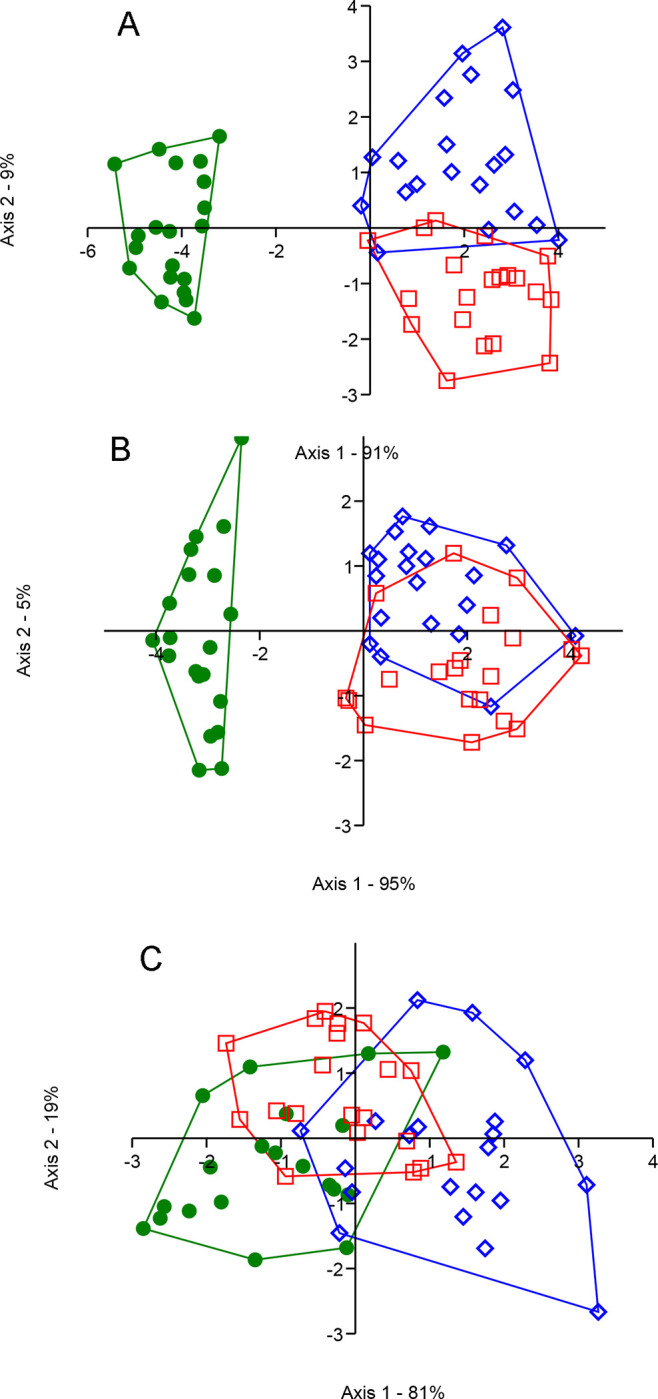
Linear discriminant analysis (LDA) based on acoustic parameters for the two subspecies and the new island mangrove population of the *Setophaga petechia* complex. (A) the 11-acoustic metrics, (B) only the metrics characteristic of phrase level, and (C) only the metrics characteristic of syllable level. *Setophaga petechia bryanti* (green), *S*. *p*. *rufivertex* (blue), and the new island mangrove population (red).

**Fig 9:**
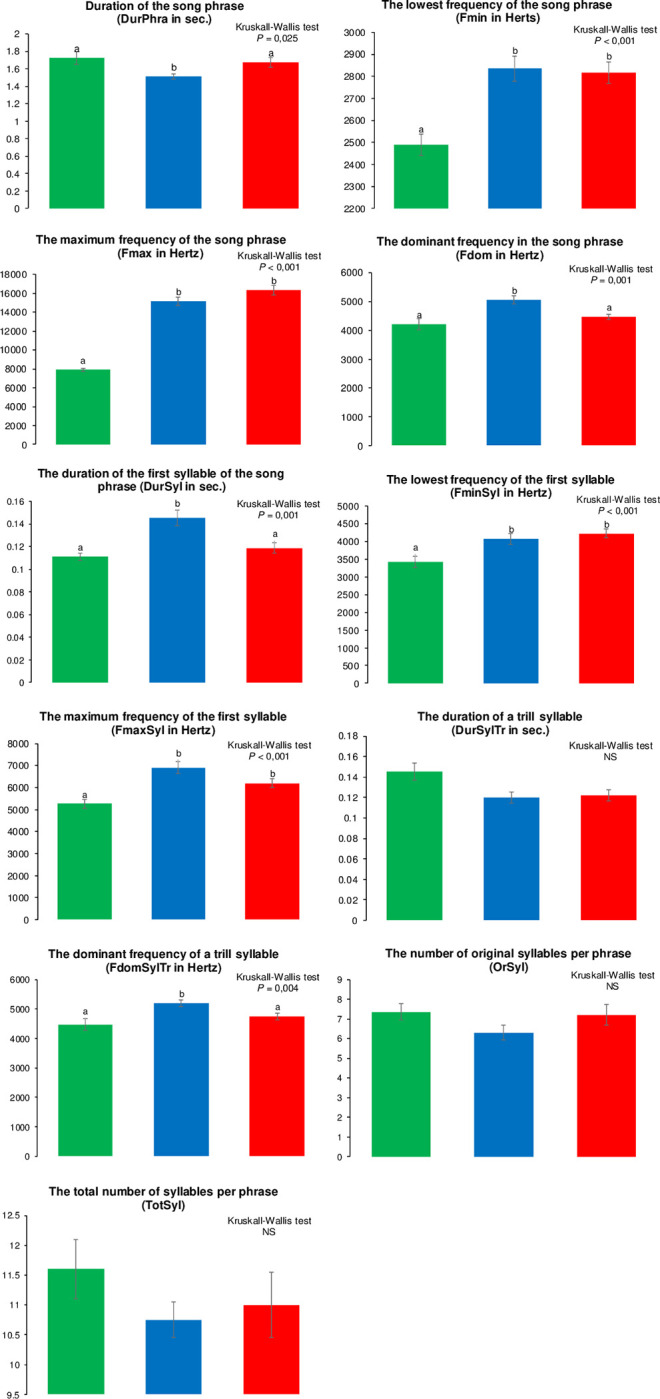
Histograms for each of the 11 acoustic metrics determined for the two subspecies and the island mangrove population of the *Setophaga petechia* complex. *Setophaga petechia bryanti* (green), *S*. *p*. *rufivertex* (blue), and the new island mangrove population (red). Mean and standard error are presented. Letters above each box represent intergroup differences follow by the Dunn post-hoc test.

The PERMANOVA analyses showed highly significant differences between the three populations for all analytical situation (all variables: *F* = 77.3, DF = 2, *P* = 0.0001; variables of syllable level: *F* = 10.66, DF = 2, *P* = 0.0001; variables of phrase level: *F* = 109.9, DF = 2, *P* = 0.0001) except for song complexity parameters (*F* = 1.25, DF = 2, *P* = 0.285) which can be observed with the LDA graphs (Fig 8A–8C). When all acoustic variables and variable characteristic of the phrase level were considered the first discriminant axis highlighted a significant (S8 Table) separation between mainland (negative side; green color in Fig 8A and 8B) and island (positive side; red and blue colors in Fig 8A and 8B) populations. Nevertheless, only the use of all variables (Fig 8A) permits the significant separation of both island populations, whereas the phrase level variables don’t (Fig 8B and S8 Table). Although the acoustic parameters of syllable level permit a significant separation (S8 Table) of the three populations, the LDA graph (Fig 8C) shows an overlap area among them. Finally, statistical analysis of acoustic parameters showed that most of the variables are significantly different among the three populations except the duration of a trill syllable (DurSylTr) and the song complexity/continuity (OrSyl, TotSyl) variables (Fig 9). The frequency variables generally highlighted a significant higher value for island populations (Fig 9). Difference between songs of the three populations can be appreciated in an audio presented in S1 Audio.

## Discussion

### Body and bill size approach

One of the most important features for reproductive isolation in birds is the morphology, another one being the song [5, 10]. Our study highlights a significant separation between the two subspecies and the new island mangrove population of *Setophaga petechia* based on the combination of measurements. A blend of the following two important conditions could lead to this separation: the isolation of populations on an island *vs* the mainland counterpart, and the habitat (mangroves *vs* coastal dunes and secondary vegetation) which conditions the foraging and the maneuverability of individuals [in 5]. It is largely documented that passerine birds respond to the “island syndrome” for which both body size and bill size for insular birds are greater than mainland counterparts [references in 62–64]. Different hypotheses have been advanced to explain this insular rule as ecological niche expansion, broader feeding niche leading to generalist foraging, reduced predation pressure and limited dispersal, among others [review in 62]. Our data partially respond to this rule. The comparison between mainland subspecies, *S*. *p*. *bryanti* living in a mangrove ecosystem and the new island population also living in mangrove generally showed greater body and bill width measurements for the island birds. Nevertheless, even though the wing length is smaller for the new island population this difference is not significant, whereas the tarsus length, bill length, and bill height showed a significantly smaller value for the new island mangrove individuals. On the other hand, it has been shown that a similar pattern of the “island syndrome” could occur for passerine birds between ecosystems as mangrove *vs* non-mangrove where mangrove populations have larger bills than relatives in non-mangrove [65, 66]. Our data corroborate very well with this hypothesis where all body and bill measurements are larger (not always significant) in the island mangrove population than in the non-mangrove island subspecies, *S*. *p*. *rufivertex*. Finally, our morphological results significantly point to the presence of the new population on Cozumel Island for which the incipient isolation process seems faster considering the habitat specificity (mangrove *vs* non-mangrove) combined with the geographical isolation (island *vs* mainland).

The multivariate analysis shows that the mainland subspecies, *S*. *p*. *bryanti*, presents a greater morphological variation than the island ones suggesting a larger ecological niche [6]; although, phenotypic plasticity or selection process are also possible hypotheses [6] more studies are needed to refine theses hypotheses. Although both island populations occupy areas in close geographical proximity, their habitat preference has probably decreased the competition for resources and permitted a rapid increase in morphological divergence between them, particularly for wing and tail length that are important in foraging behavior [references in 11].

### Phenotypic characterization

Intraspecific variation in plumage color is reported in approximately 3.5% of extant bird species [67]. Different studies have investigated the phenotypic variation in Yellow Warbler to understand origin and significance of this variation [e.g., 36, 37, 68–71]. Our study highlights an evident separation between the two subspecies and the new island mangrove population through the multivariate analysis. The first interesting characteristic observed in the new island mangrove population is the head coloration that often presents intermediate coloration between both subspecies with only chestnut-colored crown and throat, while subspecies present a completely chestnut-colored head for *S*. *p*. *bryanti* and only the chestnut-colored crown for *S*. *p*. *rufivertex*. Male plumage coloration in birds is largely accepted as an important factor for mate choice and species recognition. The presence of individuals with a new phenotype on the island could have caused a series of cascading events finalizing in reproductive isolation [72, 73]. It has been demonstrated that sexual selection, principally by females that showed preference for a new intermediate phenotype, could accelerate divergence in male plumage leading to an isolation process [references in 73]. This process leads to reproductive isolation, whether complete or not, probably under a prezygotic isolation [74]. Thus, the phenotype observed for the new mangrove individuals could be the result of this sequence of events reinforced by ecological isolation on the island from *S*. *p*. *rufivertex* through selection of different habitats. For example, it was observed the presence and reproduction of individuals of the new mangrove population in the RAMSAR site “Manglares y Humedales del Norte de Isla Cozumel”, with almost exclusively mangrove habitat, where *S*. *p*. *rufivertex* was never observed [25] that may have strengthened the process of isolation.

Another interesting phenotypic character is the width of the breast streaks. Both subspecies present a typical pattern for the streaked breast, with all mainland individuals of *S*. *p*. *bryanti* having a thinly streaked breast and all island individuals of *S*. *p*. *rufivertex* having a heavily streaked breast. In this case, all individuals of the island mangrove population present the same pattern as the island subspecies. Yellow Warbler species present a variety in the amount of brown breast streaking which is associated with different reproductive strategies [35]. Males with a heavily streaked breast are more aggressive, more territorial, and have an extra-pair mating success behavior which is important for sexual selection [35, 36, 68]. Males with thinly streaked breasts are less territorial but invest more energy in parental care [35, 36, 68]. Furthermore, it has been suggested that males with more intense colors indicate higher quality individuals [75], and that, in small populations, isolation takes place at a rapid rate [76]. This, combined with the individual advantage of the more intensely streaked breast could have led to a rapid increase in the reproductive success of the new mangrove population on the island. A Previous study [77] showed that morphological differentiation in recently colonized island passerine birds could be explained by directional selection; nevertheless, we need more time and analysis (e.g., genomic) to bring a hypothesis from these first interesting observations.

Our results, regarding the presence or absence of back-colored lines, clearly showed a preference for the absence of the back line for the three populations. Few studies propose an evolutionary explanation for this phenotypic character [78].

### Genetic perspective

Using three different methods of population aggregation, our study points to a clear separation between the three resident populations of the *Setophaga* p*etechia* complex and highlights some mixed individuals showing that reproductive isolation is not complete. Additionally, we evidenced an intermediate level of genetic diversity for the three populations of *Setophaga petechia* in Quintana Roo.

Our results suggest an intermediate level of genetic diversity if we compare them to other studies of *Setophaga petechia* using microsatellites [3, 79, 80], and intermediate to high if we compare to other bird studies using the same genetic marker (ISSR) [81, 82], considering that the higher value for *H*_*e*_ obtained with the dominant genetic marker can be of 0.5 [83]. Although the best model proposed by the HICKORY program (*Full*) suggesting inbreeding and population differentiation processes [82], the values of DIC and Dbar parameters are very close to the *f* = 0 model (no inbreeding) favoring a differentiation among populations rather than an inbreeding effect in them. The level of genetic diversity of both island populations is slightly higher than the mainland counterpart although not significantly. Generally, one can expect that island populations present lower genetic diversity than mainland counterparts [84, 85]. Nevertheless, this is not the rule for all taxonomic groups, particularly for bird species where a study suggested that island and mainland bird species did not present a difference in genetic variation [86]. Also, in species that can fly, such as birds, there is a less important heterozygosity difference between island and mainland birds [84]. Our results could suggest that the island subspecies, *S*. *p*. *rufivertex*, is well established with probably enough population size to maintain a high level of genetic diversity. The high capacity of dispersion most likely helps to maintain a good level of genetic diversity [in 84].

AMOVA and pairwise *Φ*_*PT*_ results highlight a higher genetic proximity between island populations than with the mainland subspecies. These results can be expected considering the geographic proximity between *S*. *p*. *rufivertex* and the new mangrove population and that even if each has a distinct habitat, both are very close in certain areas of the island permitting reproductive encounters. All population differentiation analyses showed an obvious separation among the three populations, with some introgression events principally between both island populations which is not surprisingly considering the potential of bird species to reproduce among them [review in 87] particularly in the *Setophaga* genus [20, 70, 71, 88–90]. Many factors could favor the evolution of the new mangrove population on the island as the song selection, the geographical barrier, the habitat isolation, the ecological divergence and the male-male aggression among others [19, 89, 91].

### Acoustic perspective

Analysis of the song of the three-peninsula populations of the *Setophaga petechia* complex shows that some song characteristics are more or less important in their separation. This result is not exceptional since it has been shown that birdsong is implied in important behaviors such as mate choice, conspecific recognition [in 92], defense of territory [40, 93], and also plays an important role in breeding habitat selection [94]. All these behaviors have implications regarding pre-mating isolation which could lead to a speciation process [in 61, 92, 95] and could explain the isolation of the new island mangrove population. Variation in birdsong between populations or subspecies has been shown previously [e.g., 13, 95]. These variations could evolve rapidly over time [92, 96, 97] as apparently has occurred in our study considering that the first record of a Mangrove-type Warbler date was in 2008 [25]; although, due to the size of the population estimated in 2014 and the lack of reporting of bird species in general, the population most likely existed a great deal earlier.

Considering all the 11 acoustic parameters, the three populations are well separated with a closer relationship between the two island populations than with the mainland subspecies. This is logical considering the geographical proximity of both island populations which could favor song transmission. Studies have shown that song, in some groups of birds, is learned based on contiguous neighbors [97–99] which could explain why both island populations have more similar songs than with *S*. *p*. *bryanti*. However, some “biases” in the learning allows for the specificity of species or subspecies [in 100]. Another study suggests that song is not a learned behavior from a neighboring song repertoire but highlights the importance of individual capacity to innovate song [61]. A recent study on song evolution in a *Setophaga* hybrid zone highlights a more complex mechanism for song production where syringeal motor gesturing (contributes to syllable morphology) could be principally learned while the respiratory patterning (contribute to song rhythm) could be more innate [101]. In our study, geographical isolation between the *Setophaga* island populations and the mainland subspecies clearly contributes to the song separation between them as demonstrated previously [13, 92, 102], which may have contributed to reproductive isolation [in 92, 103].

When we look at the syllable level, the linear discriminant analysis shows a high level of overlap among the three populations along with a higher dispersion of individual points. It has been suggested that the song characteristics associated more specifically to syllables are more representative of individual differentiation [61]. This suggests a low level of individual specificity [61] for *Setophaga petechia* in our study. To the contrary, the LDA analysis of the phrase level of the song clearly shows the separation of the island populations from the mainland subspecies, but not between both island populations. These song characteristics are more associated with the species or population differentiation [61] highlighting the important effect of the geographic separation between mainland and island populations and suggesting a faster differentiation for phrase level characteristics of song within the *Setophaga* complex. An important observation is highlighted in our study in that we have two important parameters of population differentiation: the geographical distance (island *vs* mainland) and the habitat (mangrove *vs* non-mangrove). Two kinds of song characteristics (the duration and the dominant frequency of the song phrase, and the duration of the first syllable and the dominant frequency of the trill in the first syllable of the phrase) are significantly different between mangrove inhabitants (*S*. *p*. *bryanti* on the mainland and the new mangrove population on the island) and the non-mangrove subspecies (*S*. *p*. *rufivertex* on the island). This suggests that there is no geographical distance influence but rather high and rapid evolution between populations living closer to each other but in different habitats. Some hypotheses could be suggested based on a previous study [61], as the duration of the first syllable and the importance of the trill in the individual recognition and for alarm/churr-call make them important for a rapid individual identity for neighboring subspecies or populations. Also, our results of dominant frequency show lower values for mangrove populations, as previously shown for *S*. *p*. *bryanti* compared to *S*. *p*. *aestiva* [40], according to the acoustic adaptation to environment where higher frequency are more attenuated in dense vegetation or a closed habitat (favoring low frequency) than in open habitat (favoring high frequency) [13, 40, 104]. On the other hand, two other kinds of song characteristics (lowest and maximum frequencies of the song phrase and of the first syllable) are significantly different between the mainland subspecies and the two island populations suggesting a geographical effect on these variables and a low level of evolution for the separation of neighboring populations. Finally, our results suggest no differentiation of the overall repertoire size and complexity. Generally, for passerine birds, the repertoire size is small to moderate (from less than five to ten syllables) [in 61].

## Conclusion

Our multi-character approach using morphology, phenotypic, genetic, and acoustic data permit highlighting the similarities and dissimilarities between a new mangrove population of *Setophaga petechia* on Cozumel Island with the two well-established subspecies *S*. *p*. *bryanti* and *S*. *p*. *rufivertex*. We have shown the existence of the new specific mangrove population on Cozumel Island. The question that arises now lies in its origin. Different hypotheses can be put forward; however, our results do not yet allow to point to a specific one. One hypothesis would be the result of a hybridization process that would have taken place following a chance encounter between certain individuals from the mainland (*S*. *p*. *bryanti*), the closest by far of this subspecies and the island subspecies (*S*. *p*. *rufivertex*) as suggested previously [25]. Another hypothesis would be a founder effect from the island subspecies to a new mangrove environment due to habitat disturbance affecting the species food source. What seems clear, is that this new population presents a high level of differentiation. Only a long-range study with continuous follow-up of this new population will provide insight into its evolutionary process.

## Supporting information

S1 Fig Plumage coloration variations of adults of the new island mangrove population. Initial chestnut coloration starts in the crown and the throat, with streaking reaching the base of the bill, as well as in the cheek below the eye (examples in C9-C4-C12). In individuals with almost completely colored chestnut heads (C8-C1), the crown area is darker than the rest of the head and the majority of those photographed have yellow lores and yellowish behind the eye. Photo credit: Waldemar Santamaria Rivero.
(PDF)

S2 Fig Results from Bayesian analysis to determine the best number of new clusters (*K*) with STRUCTURE software. Results obtained from ten replicates for values of *K* from 1 to 6. **(A)** Ln *P(D)* method, and **(B)** Δ*K* method follow STRUCTURE HARVEST website.
(PDF)

S1 Table Inter simple sequence repeat (ISSR) genetic markers used in analyses for the yellow warbler, *Setophaga petechia* complex. %GC: percentage of guanine and cytosine content, Ta: annealing temperature, N bands: total number of bands per primers over all samples, size: maximum range size of the DNA fragments for each primer. Following designations were used for degenerated sites: B (C or T) and D (A or T).
(PDF)

S2 Table Results of the non-parametric MANOVA analysis for morphological data. *P* value from the pairwise comparison test of the PERMANOVA between the three populations of the yellow warbler complex, *Setophaga petechia*.
(PDF)

S3 Table SIMPER analysis considering morphometric measures for the two subspecies and the new island population of the yellow warbler complex, *Setophaga petechia*. Average dissimilarity (Av. dissim), percentage of contribution for difference between groups (Contrib. %), number between parenthesis correspond to the overall average dissimilarity, red values highlight the most important contributions.
(PDF)

S4 Table DIC statistics obtained for three models implemented in HICKORY v1.1 program applied to ISSR nuclear genetic markers for the yellow warbler complex, *Setophaga petechia*. Red values show the lowest values that indicate the best model.
(PDF)

S5 Table Pairwise genetic differentiation (*Φ*_*PT*_) for the two subspecies and the new island population of the yellow warbler complex, *Setophaga petechia*, determined using GenAlex. *Φ*_*PT*_ values below diagonal and probability *P* based on 9,999 permutations above diagonal.
(PDF)

S6 Table Hierarchical analysis of molecular variance (AMOVA) based on ISSR nuclear genetic markers testing three group scenarios for the three populations in the yellow warbler complex, *Setophaga petechia*. **Scenario 1** if *S*. *p*. *rufivertex* and the new mangrove population (both on the island) are considered closer, **scenario 2** if the mainland subspecies *S*. *p*. *bryanti* is considered closer to the new mangrove population of the island, and **scenario 3** if the mainland subspecies *S*. *p*. *bryanti* is considered closer to the island subspecies *S*. *p*. *rufivertex*. Level of significance: *** *P* < 0.001, not significant (ns). The highlighted scenario in grey shows the highest percentage of molecular variance.
(PDF)

S7 Table Membership probabilities (*Q*) of the two subspecies and the new island mangrove population in each of the new clusters for *K* = 2 and *K* = 3. The highest value assigned of a population to one of the new clusters is indicated in bold.
(PDF)

S8 Table Results of the non-parametric MANOVA analysis for acoustic dataset. *P* value from the pairwise comparison test of the PERMANOVA between the three populations of the yellow warbler complex, *Setophaga petechia*.
(PDF)

S1 Audio Illustration of songs from the two subspecies and the new island mangrove population of the yellow warbler, *Setophaga petechia*. (PPTX)

S1 Data Dataset for genetic, morphometric, acoustic, and phenotypic information. (PDF)
