# Supplementary figures and images for: Multi-character approach reveals a new mangrove population of the Yellow Warbler complex, Setophaga petechia, on Cozumel Island, Mexico
Source: PLoS One. 2023 Jun 22;18(6):e0287425. doi: 10.1371/journal.pone.0287425 (PMC10287016; doi:10.1371/journal.pone.0287425)

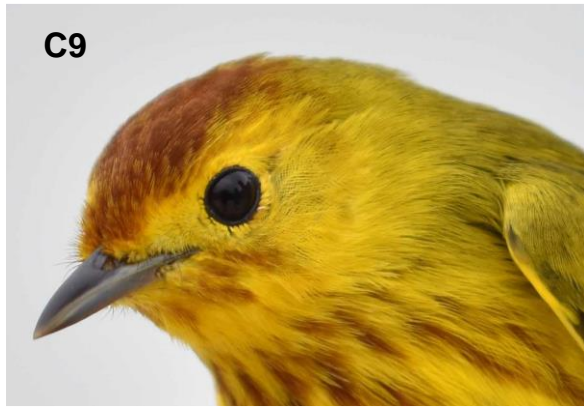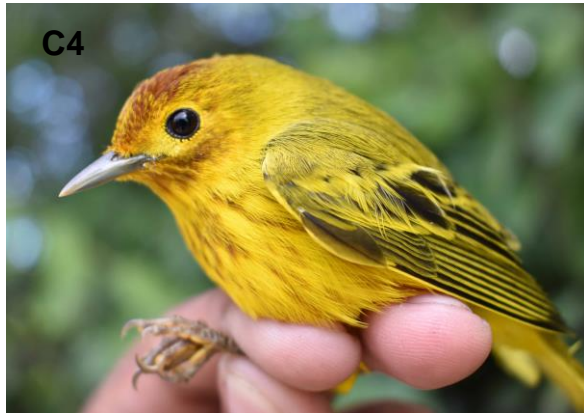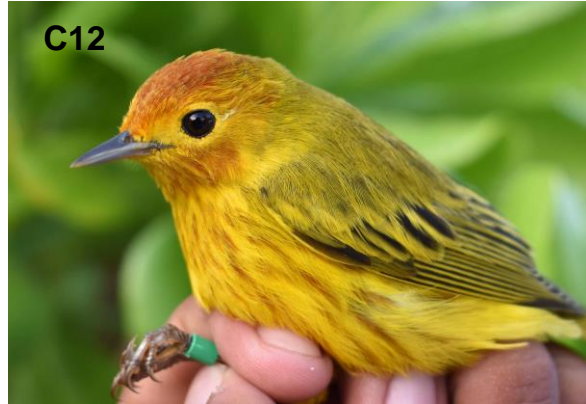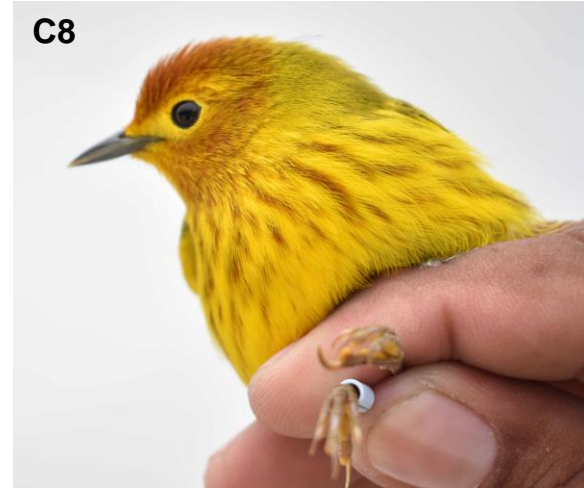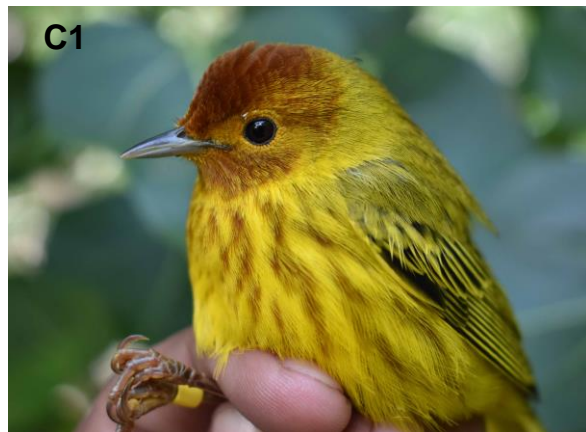

Supplement: S1 Fig — Initial chestnut coloration starts in the crown and the throat, with streaking reaching the base of the bill, as well as in the cheek below the eye (examples in C9-C4-C12). In individuals with almost completely colored chestnut heads (C8-C1), the crown area is darker than the rest of the head and the majority of those photographed have yellow lores and yellowish behind the eye. Photo credit: Waldemar Santamaria Rivero. (PDF) [file pone.0287425.s001.pdf]

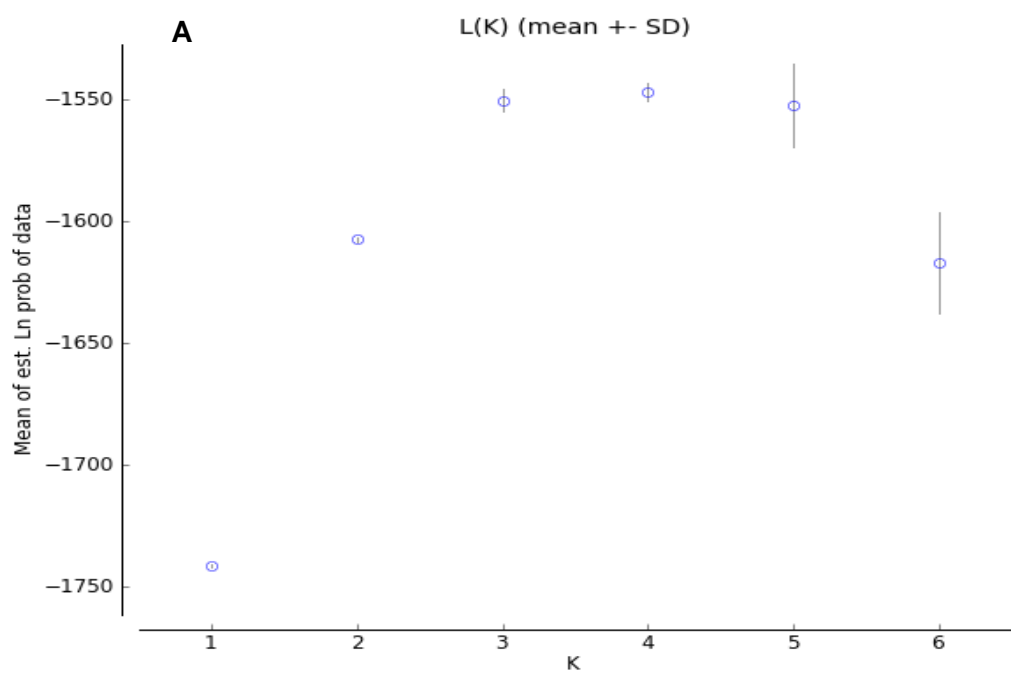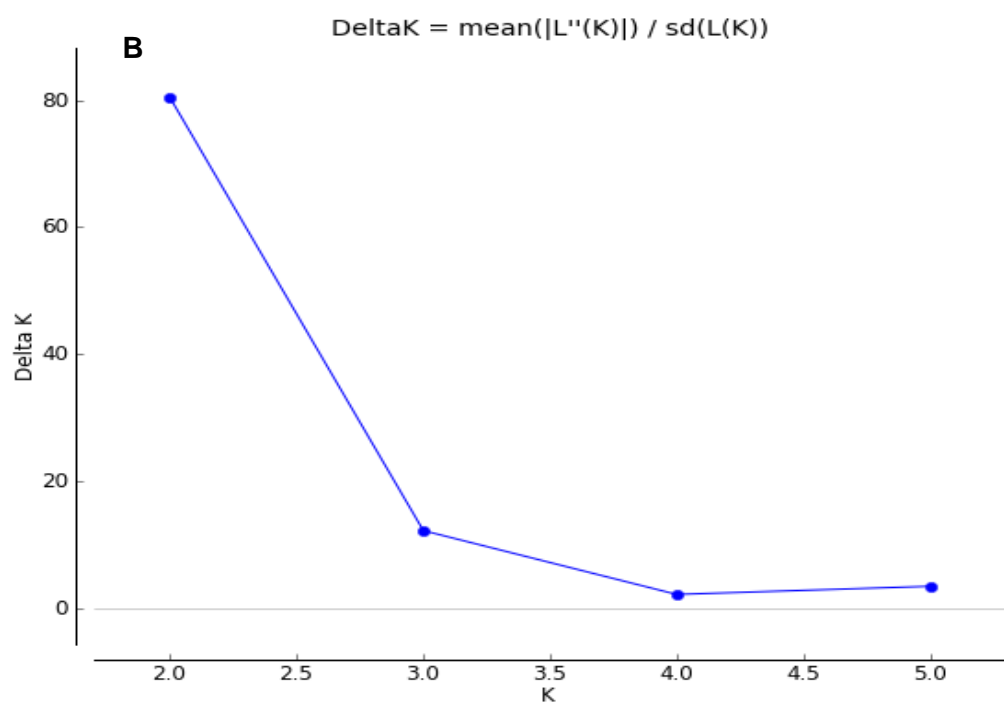

Supplement: S2 Fig — Results obtained from ten replicates for values of K from 1 to 6. (A) Ln P(D) method, and (B) ΔK method follow STRUCTURE HARVEST website. (PDF) [file pone.0287425.s002.pdf]
