# Supplementary material for: Multi-character approach reveals a new mangrove population of the Yellow Warbler complex, Setophaga petechia, on Cozumel Island, Mexico
Source: PLoS One. 2023 Jun 22;18(6):e0287425. doi: 10.1371/journal.pone.0287425 (PMC10287016; doi:10.1371/journal.pone.0287425)
Supplement: S1 Table — %GC: percentage of guanine and cytosine content, Ta: annealing temperature, N bands: total number of bands per primers over all samples, size: maximum range size of the DNA fragments for each primer. Following designations were used for degenerated sites: B (C or T) and D (A or T). (PDF) [file pone.0287425.s003.pdf]

**Primer code**	**Primer Sequences (5'→ 3')**	**%GC**	**T*a* (°C)**	***N* bands**	**Size (bp)**
$(AC)_8C$	ACACACACACACACACC	52.9	61	11	300-2000
$(AG)_8C$	AGAGAGAGAGAGAGAGC	52.9	57	13	200-1500
$BDB(ACA)_5$	BDBACAACAACAACAACA	33.3	57	10	300-2000
$(GAG)_5GC$	GAGGAGGAGGAGGAGGC	70.6	63	10	300-1500
$(GGTA)_4$	GGTAGGTAGGTAGGTA	50	52.5	14	300-2000
