# Supplementary material for: Multi-character approach reveals a new mangrove population of the Yellow Warbler complex, Setophaga petechia, on Cozumel Island, Mexico
Source: PLoS One. 2023 Jun 22;18(6):e0287425. doi: 10.1371/journal.pone.0287425 (PMC10287016; doi:10.1371/journal.pone.0287425)
Supplement: S2 Table — P value from the pairwise comparison test of the PERMANOVA between the three populations of the yellow warbler complex, Setophaga petechia. (PDF) [file pone.0287425.s004.pdf]

	S. p. bryanti	***S. p. rufivertex***	**New island population**
S. p. bryanti		0.0009	0.0138
S. p. rufivertex	0.0009		0.0003
**New island population**	0.0138	0.0003	
