# Supplementary material for: Multi-character approach reveals a new mangrove population of the Yellow Warbler complex, Setophaga petechia, on Cozumel Island, Mexico
Source: PLoS One. 2023 Jun 22;18(6):e0287425. doi: 10.1371/journal.pone.0287425 (PMC10287016; doi:10.1371/journal.pone.0287425)
Supplement: S3 Table — Average dissimilarity (Av. dissim), percentage of contribution for difference between groups (Contrib. %), number between parenthesis correspond to the overall average dissimilarity, red values highlight the most important contributions. (PDF) [file pone.0287425.s005.pdf]

	Av. dissim	Contrib. %	Cumulative %
	S.p.bryanti / *S.p.rufivertex* / New island population (73.68)		
**Total length**	38.93	52.83	52.83
**Wing length**	19.79	26.86	79.7
**Tail length**	11.45	15.54	95.24
**Tarsus length**	2.424	3.29	98.53
**Bill length**	0.8428	1.144	99.67
**Bill height**	0.1503	0.204	99.87
**Bill width**	0.0923	0.1253	100
	S.p.bryanti / *S.p.rufivertex* (82.06)		
**Total length**	33.12	40.36	40.36
**Wing length**	32.17	39.2	79.56
**Tail length**	12.13	14.77	94.34
**Tarsus length**	3.206	3.907	98.25
**Bill length**	1.182	1.441	99.69
**Bill height**	0.1583	0.1929	99.88
**Bill width**	0.09827	0.1198	100
	S.p.bryanti / New island population (67.87)		
**Total length**	40.93	60.31	60.31
**Wing length**	12.97	19.11	79.42
**Tail length**	9.928	14.63	94.05
**Tarsus length**	2.908	4.285	98.33
**Bill length**	0.8766	1.292	99.62
**Bill height**	0.1375	0.2026	99.82
**Bill width**	0.1189	0.1752	100
	S.p.rufivertex / New island population (72.09)		
**Total length**	41.93	58.17	58.17
**Wing length**	15.79	21.91	80.07
**Tail length**	12.31	17.08	97.15
**Tarsus length**	1.31	1.818	98.97
**Bill length**	0.525	0.7282	99.7
**Bill height**	0.1556	0.2159	99.91
**Bill width**	0.06223	0.08632	100
