# Supplementary material for: Multi-character approach reveals a new mangrove population of the Yellow Warbler complex, Setophaga petechia, on Cozumel Island, Mexico
Source: PLoS One. 2023 Jun 22;18(6):e0287425. doi: 10.1371/journal.pone.0287425 (PMC10287016; doi:10.1371/journal.pone.0287425)
Supplement: S4 Table — Red values show the lowest values that indicate the best model. (PDF) [file pone.0287425.s006.pdf]

Models	DIC	Dbar	Dhat	pD
Full	634.2	536.7	436.1	100.6
f = 0	647.6	537.3	427.0	110.3
θ = 0	1047.4	1002.4	957.4	45.0
