# Supplementary material for: Multi-character approach reveals a new mangrove population of the Yellow Warbler complex, Setophaga petechia, on Cozumel Island, Mexico
Source: PLoS One. 2023 Jun 22;18(6):e0287425. doi: 10.1371/journal.pone.0287425 (PMC10287016; doi:10.1371/journal.pone.0287425)
Supplement: S5 Table — ΦPT values below diagonal and probability P based on 9,999 permutations above diagonal. (PDF) [file pone.0287425.s007.pdf]

	S. p. bryanti	*S. p. rufivertex*	**New island population**
S. p. bryanti		< 0.001	< 0.001
S. p. rufivertex	0.251		< 0.001
**New island population**	0.223	0.155	
