# Supplementary material for: Multi-character approach reveals a new mangrove population of the Yellow Warbler complex, Setophaga petechia, on Cozumel Island, Mexico
Source: PLoS One. 2023 Jun 22;18(6):e0287425. doi: 10.1371/journal.pone.0287425 (PMC10287016; doi:10.1371/journal.pone.0287425)
Supplement: S6 Table — Scenario 1 if S. p. rufivertex and the new mangrove population (both on the island) are considered closer, scenario 2 if the mainland subspecies S. p. bryanti is considered closer to the new mangrove population of the island, and scenario 3 if the mainland subspecies S. p. bryanti is considered closer to the island subspecies S. p. rufivertex. Level of significance: *** P < 0.001, not significant (ns). The highlighted scenario in grey shows the highest percentage of molecular variance. (PDF) [file pone.0287425.s008.pdf]

	df	% variation	*Phi* values	*p*
**Scenario 1**				
Among groups	1	7	Phi_{RT}: 0.074	***
Among subspecies/New island population - within groups	1	16	Phi_{PR}: 0.168	***
Within subspecies/ New island population	57	77	Phi_{PT}: 0.229	***
**Scenario 2**				
Among groups	1	0	Phi_{RT}: 0.001	ns
Among subspecies/ New island population - within groups	1	21	Phi_{PR}: 0.209	***
Within subspecies/ New island population	57	79	Phi_{PT}: 0.210	***
**Scenario 3**				
Among groups	1	0	Phi_{RT}: 0.000	ns
Among subspecies/ New island population - within groups	1	25	Phi_{PR}: 0.248	***
Within subspecies/ New island population	57	75	Phi_{PT}: 0.189	***
