# Supplementary material for: Multi-character approach reveals a new mangrove population of the Yellow Warbler complex, Setophaga petechia, on Cozumel Island, Mexico
Source: PLoS One. 2023 Jun 22;18(6):e0287425. doi: 10.1371/journal.pone.0287425 (PMC10287016; doi:10.1371/journal.pone.0287425)
Supplement: S7 Table — The highest value assigned of a population to one of the new clusters is indicated in bold. (PDF) [file pone.0287425.s009.pdf]

	**Two clusters (*K* = 2)**		**Three clusters (*K* = 3)**		
	1	2	1	2	3
S. p. bryanti	**0.912**	0.088	**0.876**	0.055	0.069
S. p. rufivertex	0.052	**0.948**	0.038	**0.828**	0.134
**New island population**	0.073	**0.927**	0.039	0.101	**0.860**
