# Supplementary material for: Multi-character approach reveals a new mangrove population of the Yellow Warbler complex, Setophaga petechia, on Cozumel Island, Mexico
Source: PLoS One. 2023 Jun 22;18(6):e0287425. doi: 10.1371/journal.pone.0287425 (PMC10287016; doi:10.1371/journal.pone.0287425)
Supplement: S8 Table — P value from the pairwise comparison test of the PERMANOVA between the three populations of the yellow warbler complex, Setophaga petechia. (PDF) [file pone.0287425.s010.pdf]

	S. p. bryanti	*S. p. rufivertex*	**New island population**
	All acoustic variables		
S. p. bryanti		0.0001	0.0001
S. p. rufivertex	0.0001		0.0353
**New island population**	0.0001	0.0353	
	Acoustic variables characteristic of phrase level		
S. p. bryanti		0.0001	0.0001
S. p. rufivertex	0.0001		0.0655
**New island population**	0.0001	0.0655	
	Acoustic variables characteristic of syllable level		
S. p. bryanti		0.0001	0.0001
S. p. rufivertex	0.0001		0.0162
**New island population**	0.0014	0.0162	
