# Supplementary material for: Multi-character approach reveals a new mangrove population of the Yellow Warbler complex, Setophaga petechia, on Cozumel Island, Mexico
Source: PLoS One. 2023 Jun 22;18(6):e0287425. doi: 10.1371/journal.pone.0287425 (PMC10287016; doi:10.1371/journal.pone.0287425)
Supplement: S1 Audio — (PPTX) [file pone.0287425.s011.pptx]

## Slide 1
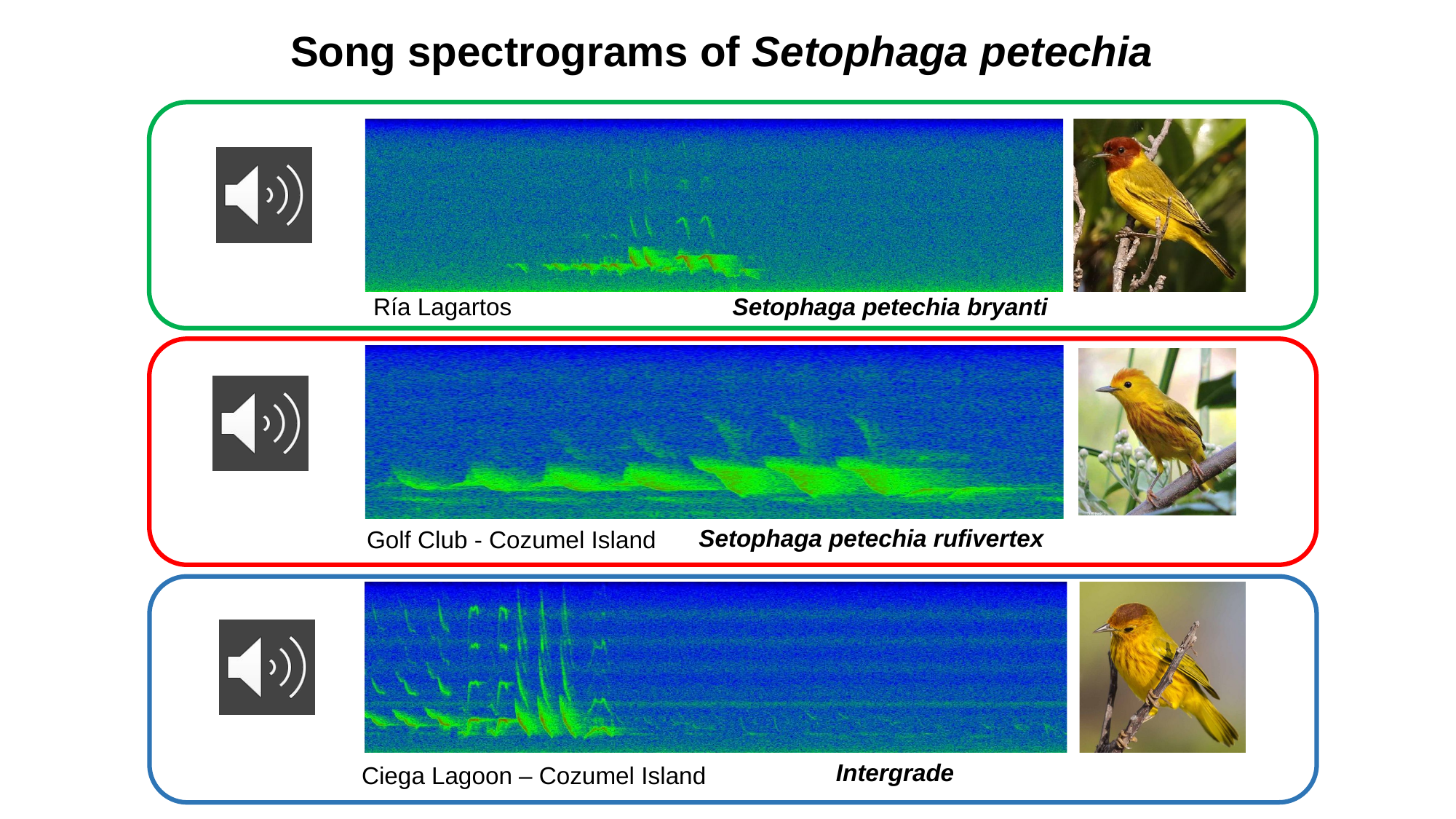

Song spectrograms of Setophaga petechia
Ría Lagartos
Setophaga petechia bryanti
Setophaga petechia rufivertex
Golf Club - Cozumel Island
Intergrade
Ciega Lagoon – Cozumel Island
