# Supplementary material for: Multi-character approach reveals a new mangrove population of the Yellow Warbler complex, Setophaga petechia, on Cozumel Island, Mexico
Source: PLoS One. 2023 Jun 22;18(6):e0287425. doi: 10.1371/journal.pone.0287425 (PMC10287016; doi:10.1371/journal.pone.0287425)
Supplement: S1 Data — (PDF) [file pone.0287425.s012.pdf]

MORPHOMETRIC DATA

Groups	Sex	Wing length	Tail length	Tarsus length	Total length	Bill length	Bill width	Bill height	Weight
S. p. bryanti	macho	66.00	50.00	22.80	120.00	9.20	1.30	1.90	13.20
S. p. bryanti	macho	66.00	51.00	22.60	122.00	8.70	1.80	1.90	13.40
S. p. bryanti	macho	63.00	48.00	21.90	117.00	8.10	2.00	1.80	13.40
S. p. bryanti	macho	63.00	50.00	21.90	119.00	9.20	1.20	1.60	12.20
S. p. bryanti	hembra	65.00	52.00	21.70	130.00	9.80	1.30	1.80	13.60
S. p. bryanti	hembra	61.00	46.00	21.70	121.00	9.90	1.70	2.00	13.30
S. p. bryanti	macho	67.00	51.00	22.50	127.00	9.00	1.80	2.00	13.40
S. p. bryanti	hembra	61.00	47.00	22.90	120.00	9.70	1.90	1.80	12.30
S. p. bryanti	macho	66.00	52.00	21.70	130.00	8.90	1.90	1.80	12.90
S. p. bryanti	macho	67.00	52.00	21.00	131.00	8.00	2.00	1.60	12.40
S. p. bryanti	hembra	61.00	50.00	21.50	124.00	8.40	1.90	1.60	11.30
S. p. bryanti	macho	66.00	49.00	22.00	124.00	9.00	2.00	1.70	11.80
S. p. bryanti	hembra	64.00	50.00	21.50	120.00	8.00	1.90	1.70	10.40
S. p. bryanti	hembra	61.00	45.00	20.00	120.00	8.00	1.90	1.70	12.20
S. p. bryanti	macho	68.00	52.00	21.00	127.00	9.00	1.90	1.70	10.40
S. p. bryanti	hembra	60.00	45.00	21.00	129.00	7.90	1.80	1.70	10.50
S. p. rufivertex	macho	60.00	50.00	20.00	125.00	8.70	1.90	1.20	9.80
S. p. rufivertex	hembra	58.00	46.00	18.90	120.00	8.40	1.50	1.00	9.30
S. p. rufivertex	macho	61.00	50.00	21.90	125.00	7.90	1.90	1.80	9.70
S. p. rufivertex	macho	59.00	48.00	21.50	120.00	7.90	1.90	1.60	10.60
S. p. rufivertex	macho	59.00	49.00	20.00	122.00	8.70	1.90	1.60	9.10
S. p. rufivertex	macho	57.00	46.00	20.00	116.00	7.00	1.70	1.50	8.50
S. p. rufivertex	hembra	58.00	50.00	20.00	124.00	8.80	2.00	1.80	10.70
S. p. rufivertex	macho	59.00	42.00	20.00	115.00	7.00	1.60	1.00	8.30
S. p. rufivertex	macho	58.00	50.00	21.00	125.00	9.00	1.80	1.60	10.20
S. p. rufivertex	macho	58.00	47.00	19.80	120.00	8.00	2.00	1.60	9.70
S. p. rufivertex	macho	60.00	46.00	20.00	125.00	8.00	2.00	1.80	8.90
S. p. rufivertex	macho	61.00	51.00	20.50	126.00	8.70	2.00	1.00	10.00
S. p. rufivertex	macho	59.00	51.00	19.90	123.00	8.00	1.70	1.80	9.20
S. p. rufivertex	macho	59.00	49.00	20.00	123.00	8.00	2.00	1.60	9.90
S. p. rufivertex	macho	59.00	51.00	20.00	129.00	8.00	2.00	1.60	9.40
S. p. rufivertex	macho	60.00	49.00	20.00	122.00	8.00	1.90	1.70	9.20
S. p. rufivertex	macho	60.00	51.00	20.70	125.00	8.00	2.00	1.50	9.90
Island mangrove	macho	62.00	51.00	21.20	131.00	8.00	1.60	1.60	12.10
Island mangrove	macho	61.00	50.00	20.80	125.00	9.00	2.00	1.30	10.90
Island mangrove	macho	59.00	47.00	20.00	123.00	8.00	1.40	1.50	9.90
Island mangrove	macho	62.00	52.00	20.00	127.00	8.00	2.00	1.60	11.30
Island mangrove	macho	60.00	51.00	19.70	123.00	7.70	1.90	1.50	9.30
Island mangrove	macho	61.00	52.00	19.90	127.00	9.00	2.00	1.00	9.10
Island mangrove	macho	61.00	50.00	18.00	124.00	8.00	1.40	1.00	9.20
Island mangrove	macho	62.00	52.00	21.00	131.00	8.80	2.00	1.80	10.00
Island mangrove	macho	60.00	49.00	20.30	125.00	8.00	2.00	1.70	9.90
Island mangrove	macho	62.00	50.00	20.00	122.00	8.00	1.80	1.60	9.80
Island mangrove	macho	61.00	49.00	20.00	123.00	8.50	2.00	1.60	10.10
Island mangrove	macho	66.00	52.00	21.00	130.00	9.00	2.00	1.70	10.50
Island mangrove	macho	63.00	46.00	20.00	124.00	8.00	1.90	1.60	10.40
Island mangrove	macho	65.00	51.00	20.90	126.00	8.00	2.00	1.80	10.60
Island mangrove	hembra	64.00	51.00	20.00	126.00	8.00	2.00	1.00	10.40
Island mangrove	macho	65.00	52.00	21.80	133.00	9.00	2.00	1.80	11.20
Island mangrove	hembra	63.00	50.00	21.90	130.00	8.70	2.00	1.80	10.20
Island mangrove	macho	65.00	53.00	21.00	128.00	8.00	2.00	1.90	11.70
Island mangrove	macho	64.00	51.00	21.60	133.00	8.00	2.00	1.80	12.20

PHENOTYPIC DATA

Chesnut crown colored	Chesnut throat colored	Complete chesnut head colored	Heavily streaked breast	Thinly streaked breast	Presence back lines	Ausence back lines	Presence tail lines	Ausence tail lines	SY	ASY
0	0	1	0	1	0	1	0	1	0	1
0	0	1	0	1	0	1	0	1	0	1
0	0	1	0	1	0	1	0	1	0	1
0	0	1	0	1	0	1	0	1	0	1
0	0	1	0	1	0	1	0	1	0	1
0	0	1	0	1	0	1	0	1	0	1
0	0	1	0	1	0	1	0	1	0	1
0	0	1	0	1	0	1	0	1	0	1
0	0	1	0	1	0	1	0	1	0	1
0	0	1	0	1	0	1	0	1	0	1
1	0	0	1	0	0	1	0	1	0	1
1	0	0	1	0	0	1	0	1	1	0
1	0	0	1	0	0	1	1	0	0	1
1	0	0	1	0	0	1	1	0	1	0
1	0	0	1	0	0	1	1	0	0	1
1	0	0	1	0	0	1	0	1	1	0
1	0	0	1	0	1	0	1	0	0	1
1	0	0	1	0	0	1	1	0	0	1
1	0	0	1	0	1	0	1	0	0	1
1	0	0	1	0	0	1	1	0	0	1
1	0	0	1	0	0	1	1	0	1	0
1	0	0	1	0	1	0	1	0	0	1
1	0	0	1	0	1	0	1	0	0	1
1	0	0	1	0	1	0	1	0	0	1
1	0	0	1	0	0	1	0	1	0	1
1	1	0	1	0	1	0	1	0	0	1
1	1	0	1	0	0	1	1	0	1	0
1	1	0	1	0	0	1	0	1	1	0
1	1	0	1	0	0	1	1	0	0	1
1	1	0	1	0	0	1	1	0	1	0
1	1	0	1	0	0	1	1	0	1	0
1	1	0	1	0	0	1	1	0	1	0
1	1	0	1	0	0	1	0	1	0	1
1	1	0	1	0	1	0	0	1	1	0
1	1	0	1	0	1	0	1	0	1	0
1	1	0	1	0	0	1	0	1	0	1
1	1	0	1	0	0	1	1	0	0	1
1	1	0	1	0	0	1	0	1	0	1
1	1	0	1	0	0	1	0	1	0	1
1	1	0	1	0	0	1	0	1	0	1
1	1	0	1	0	0	1	0	1	1	0
1	1	0	1	0	0	1	0	1	0	1

GENETIC DATA

GGTA4														(GAG)5GC										(AG)8C													BDB(ACA)5										(AC)8C									
1	0	1	1	1	0	0	1	0	0	0	0	0	0	1	1	1	1	1	0	1	0	0	1	1	1	1	1	1	0	0	0	0	0	0	1	0	1	1	1	1	0	0	0	0	0	0	1	1	1	1	0	1	1	0	0	0
1	1	1	1	1	1	1	0	0	0	0	0	1	0	1	1	1	1	1	0	1	0	0	1	1	1	1	1	1	0	0	0	0	0	0	1	0	0	0	1	1	1	0	0	0	0	0	1	1	0	1	0	1	0	1	0	1
1	1	1	1	1	1	0	1	0	1	0	0	1	0	1	1	1	1	1	0	1	1	0	1	1	1	1	1	1	0	0	0	0	1	0	1	0	1	1	1	1	1	0	0	0	0	0	1	1	1	0	0	1	0	1	0	1
1	1	1	1	1	1	1	1	1	0	0	0	0	0	1	1	1	1	1	0	1	1	0	1	1	1	1	1	1	0	0	0	0	1	1	1	0	1	1	1	1	1	0	0	0	0	0	1	1	1	1	0	1	0	0	0	1
1	1	1	1	1	1	0	1	1	0	0	0	1	0	1	1	1	1	1	1	1	1	0	1	1	1	1	1	1	0	0	0	1	1	1	1	1	1	0	1	1	0	0	0	0	0	0	1	1	1	0	1	1	0	1	0	1
1	1	1	1	1	1	0	0	1	1	0	0	0	0	1	1	1	1	1	1	1	0	1	1	1	1	1	1	1	0	0	1	0	0	1	1	0	1	1	1	1	0	0	0	0	0	0	1	1	1	1	1	1	0	0	0	0
1	1	1	0	1	1	1	1	0	1	0	0	0	0	1	1	1	1	1	1	1	1	1	1	1	1	1	1	1	0	0	1	0	1	1	1	0	1	1	1	1	0	0	0	0	0	0	1	1	1	0	0	1	0	0	0	1
1	1	1	1	1	1	0	0	0	1	0	0	0	0	1	1	1	1	1	0	0	0	0	1	1	1	1	1	1	1	1	1	1	1	1	1	1	1	1	1	1	0	0	0	0	0	0	1	1	1	0	1	1	0	0	0	1
1	1	1	0	1	1	1	0	1	1	0	0	0	0	1	1	1	1	1	1	1	1	0	1	1	1	1	1	1	0	1	0	0	1	0	1	0	0	1	1	1	0	0	0	0	0	0	1	1	1	0	0	1	0	0	0	1
1	0	1	0	1	1	1	1	1	1	0	0	0	0	1	1	1	1	1	1	1	1	0	1	1	1	1	1	1	1	0	1	1	0	1	1	0	0	1	1	1	0	0	0	0	0	0	0	1	1	0	1	1	1	0	0	1
1	1	1	0	1	1	1	0	0	0	0	0	0	0	1	1	1	1	1	1	1	1	0	1	1	1	1	1	0	1	1	1	0	1	1	1	0	1	1	1	1	1	0	0	0	0	0	1	1	1	0	1	1	1	1	0	1
1	0	1	0	1	1	1	0	1	0	0	0	0	0	1	1	1	1	1	1	1	1	0	0	1	1	1	1	1	1	1	0	1	1	1	1	0	1	1	1	1	1	0	0	0	0	0	1	1	1	0	1	1	1	0	0	0
1	1	1	0	1	1	0	1	1	0	0	0	0	0	1	1	1	1	1	1	1	1	1	1	1	1	1	1	0	0	1	0	1	1	1	1	1	1	1	1	1	0	0	0	0	0	0	1	1	1	1	0	1	0	1	0	1
1	1	1	0	0	1	0	1	1	1	0	0	0	0	1	1	1	1	1	1	0	1	0	0	1	1	1	1	1	0	0	0	1	1	1	1	0	1	1	1	1	1	0	0	0	0	0	1	1	1	0	0	1	0	1	0	1
1	1	1	0	0	1	1	1	0	1	0	0	0	0	1	1	1	1	1	1	0	1	0	0	1	1	1	1	0	1	1	0	1	1	0	1	0	1	1	1	1	1	0	0	0	0	0	1	1	1	0	0	1	0	0	0	1
1	0	1	1	1	1	0	0	0	0	0	0	0	0	1	1	1	1	1	1	1	0	0	0	1	1	1	1	1	1	1	0	1	1	1	1	0	1	1	1	1	0	0	0	0	0	0	1	1	1	0	1	1	1	1	0	0
1	1	1	0	1	1	0	0	0	0	0	0	0	0	1	1	1	1	1	1	1	1	1	1	1	1	0	1	0	1	1	1	1	1	1	1	1	1	1	1	1	0	0	0	0	0	0	1	1	1	1	0	1	0	1	0	1
1	1	1	1	1	1	0	0	0	0	0	0	0	0	1	1	1	1	1	1	1	0	0	0	1	1	0	1	1	0	0	0	0	0	0	1	0	1	1	1	1	0	0	0	0	0	0	1	1	1	1	1	0	1	0	0	1
1	0	1	0	0	1	1	0	0	1	0	0	0	0	1	1	1	1	1	0	1	0	0	0	1	1	0	1	1	0	1	1	1	1	1	1	0	1	0	1	1	0	0	0	0	0	1	1	1	1	0	1	1	0	1	0	0
1	0	0	0	1	1	1	1	1	0	0	0	0	0	1	1	1	1	1	0	0	0	0	0	1	1	0	1	0	0	1	1	1	1	1	1	0	1	1	1	1	0	0	0	1	0	0	1	1	1	1	1	1	0	0	0	1
1	0	1	1	1	0	1	0	0	1	0	0	0	0	1	1	1	1	0	1	1	0	0	0	1	1	0	1	0	1	1	0	1	0	0	1	0	1	0	1	1	1	0	1	0	0	1	1	1	0	1	1	1	0	0	1	1
1	0	1	0	1	1	1	1	1	0	0	1	0	0	1	1	1	1	1	1	1	0	0	0	1	1	0	1	0	1	1	0	0	1	1	1	0	0	0	1	1	1	1	1	0	1	1	1	1	0	1	0	0	1	0	1	1
1	1	1	1	0	0	1	0	0	0	0	0	0	0	1	1	1	1	0	1	1	0	0	0	1	1	0	1	0	1	1	1	0	0	0	1	0	0	1	1	1	1	1	1	1	1	0	0	1	1	1	0	1	1	0	0	0
1	1	0	1	1	1	0	0	0	1	0	0	0	0	1	1	1	1	0	1	1	0	0	0	1	1	0	1	1	0	1	1	1	1	0	1	0	1	1	1	1	0	1	1	1	1	0	1	1	1	1	0	1	0	0	1	1
1	1	1	0	0	1	0	1	0	0	1	0	0	1	1	1	1	1	0	0	1	0	0	0	1	1	1	1	0	1	1	1	1	0	0	1	0	1	1	1	1	1	0	1	0	0	0	1	1	1	1	1	1	0	0	0	1
1	1	0	0	0	1	1	0	0	1	1	1	1	0	1	1	1	1	1	0	0	0	0	0	1	1	0	1	1	0	0	1	1	0	0	1	0	1	1	1	1	1	1	1	0	1	0	1	1	1	1	1	0	0	1	1	1
1	1	0	1	0	1	0	0	0	1	1	1	0	0	1	1	1	1	1	0	0	0	0	0	1	1	0	1	0	1	1	0	0	1	0	1	0	1	0	1	1	0	1	1	0	1	1	1	1	1	1	1	1	0	0	1	1
1	1	1	1	0	0	1	0	0	1	0	1	1	0	1	1	1	1	1	1	1	0	0	0	1	1	0	1	0	1	1	0	1	0	0	1	0	1	0	1	1	1	0	0	0	0	1	1	1	1	1	1	1	0	0	1	1
1	1	1	1	0	0	1	0	0	1	1	1	1	0	1	1	1	1	1	1	1	0	0	0	1	1	1	1	1	1	0	0	0	0	0	1	0	0	0	1	1	1	1	0	0	0	0	1	1	1	1	0	1	0	0	1	1
1	1	1	1	0	0	0	0	0	0	1	1	0	0	1	1	1	1	1	0	0	0	0	0	1	1	0	1	0	1	1	0	1	0	0	1	0	1	1	1	1	0	1	1	1	1	0	1	1	0	1	0	1	0	1	1	0
1	1	0	1	0	0	1	1	0	0	1	0	1	0	1	1	1	1	1	0	0	0	0	0	1	1	0	1	0	1	0	1	0	0	0	1	0	1	1	1	1	1	1	1	1	1	0	1	1	0	1	1	1	0	1	1	1
1	0	1	1	0	1	0	1	0	0	0	0	1	0	1	1	1	1	1	1	1	0	0	0	1	1	1	1	0	1	1	0	1	1	0	1	0	1	0	1	1	1	1	0	0	1	0	1	1	1	1	0	1	1	0	0	1
1	1	0	1	0	1	1	0	0	0	1	1	1	0	1	1	1	1	1	1	1	0	0	0	1	1	0	1	0	1	0	0	1	0	0	1	0	1	1	1	1	1	0	0	1	0	0	1	1	1	1	1	1	0	0	0	0
1	1	1	1	0	1	0	0	0	0	0	1	0	0	1	1	1	1	0	1	1	0	0	0	1	1	0	1	0	1	1	0	0	1	0	0	0	0	1	1	1	0	1	1	1	0	0	1	1	0	1	1	0	0	0	1	1
1	1	1	0	0	0	1	1	1	0	0	1	0	0	1	1	1	1	1	1	1	0	0	0	1	1	1	1	1	0	0	1	1	0	1	1	0	0	1	1	1	1	0	0	0	0	0	0	1	0	1	1	1	1	0	1	1
1	1	0	0	0	0	1	1	0	0	0	1	0	0	1	1	1	1	0	1	1	0	0	0	1	1	0	1	1	0	1	1	0	0	0	1	0	0	1	1	1	0	1	0	0	1	0	-1	-1	-1	-1	-1	-1	-1	-1	-1	-1
1	0	0	0	0	0	0	0	0	0	0	0	0	0	1	1	1	1	0	0	0	0	0	1	1	1	0	1	0	1	1	0	1	0	0	1	0	0	1	1	1	1	1	1	1	1	1	0	1	0	1	0	1	1	0	1	0
0	1	1	0	0	0	1	0	0	0	0	0	0	1	1	1	1	1	0	0	0	0	0	1	1	1	0	1	0	1	1	0	1	0	0	1	0	0	1	1	1	1	0	0	0	0	0	1	1	0	1	0	1	0	0	0	1
1	1	0	0	1	0	1	1	0	1	0	0	0	0	1	1	1	1	0	0	0	0	0	1	1	1	0	1	0	1	1	0	0	1	1	1	1	0	0	1	1	1	0	1	1	1	0	1	1	0	1	0	1	0	1	0	0
1	1	1	0	0	0	1	1	0	1	0	0	0	0	1	1	1	1	0	0	0	0	0	1	1	1	0	1	0	1	0	1	1	0	0	1	0	1	1	1	1	1	1	0	0	0	0	1	1	1	1	0	1	0	0	0	1
0	1	0	0	0	0	1	0	0	0	0	0	0	1	1	1	1	1	1	0	1	0	0	1	1	1	1	1	1	1	1	1	0	1	1	1	0	1	1	1	1	0	0	0	1	0	1	1	1	1	0	0	1	0	0	1	0
0	0	0	1	0	1	1	0	1	1	0	0	1	1	1	1	1	1	1	0	0	0	0	1	1	1	1	1	0	1	1	0	1	0	0	1	0	1	1	1	1	1	0	0	0	0	0	1	1	1	1	0	1	0	0	1	1
1	1	1	0	1	0	1	1	1	1	0	0	0	0	1	1	1	1	1	0	0	0	0	1	1	1	1	1	0	1	1	0	1	1	1	1	1	0	1	1	1	0	1	1	1	1	1	1	1	1	1	0	1	1	0	1	0
0	0	1	1	0	1	1	0	1	1	0	0	0	1	1	1	1	1	0	0	0	0	0	1	1	1	1	1	0	1	1	1	1	1	0	1	0	0	1	1	1	0	0	0	1	0	0	0	1	1	1	0	1	1	0	0	1
0	1	1	0	0	0	1	0	1	1	0	0	0	1	1	1	1	1	1	0	0	0	0	0	1	1	0	1	0	1	1	0	1	1	0	1	0	1	1	1	1	0	0	0	0	0	0	0	1	1	0	0	1	0	1	0	0
0	1	1	0	0	0	0	0	0	0	0	0	0	0	1	1	1	1	1	0	1	0	0	0	1	1	1	1	0	1	1	1	1	1	1	1	0	1	1	1	1	0	0	0	1	1	0	1	1	1	0	0	0	1	1	0	0
1	1	1	1	0	0	1	1	1	0	0	0	0	0	1	1	1	1	1	0	1	0	0	0	1	1	1	1	0	1	1	0	0	1	1	1	1	0	1	1	1	0	1	0	0	1	0	1	1	1	0	0	1	0	0	0	0
0	1	0	0	0	0	1	1	1	0	0	0	0	1	1	1	1	1	1	1	0	0	0	1	1	1	0	1	0	1	1	1	0	1	1	1	0	0	1	1	1	0	0	0	0	1	0	1	1	1	0	0	1	0	0	0	1
1	1	0	1	0	0	1	1	0	0	0	0	0	0	1	1	1	1	1	0	1	0	0	1	1	1	0	1	0	1	0	0	1	0	0	1	0	1	0	1	1	1	0	1	0	1	0	1	1	1	0	0	1	1	0	0	0
1	1	0	0	0	0	1	1	1	0	0	0	0	0	1	1	1	1	1	0	1	0	0	0	1	1	1	1	0	1	0	0	1	0	0	1	0	1	0	1	1	0	1	1	1	1	0	1	1	1	1	0	1	1	0	0	0
0	1	1	0	0	0	1	1	1	1	0	0	0	1	1	1	1	1	1	1	1	0	0	0	1	1	1	1	0	1	1	0	1	1	1	1	0	1	0	1	1	0	0	0	0	0	0	1	1	1	0	1	1	1	0	1	0
0	0	1	0	0	0	1	1	1	1	0	0	0	1	1	1	1	1	1	0	1	0	0	0	1	1	1	1	0	1	1	1	0	1	0	1	0	0	1	1	1	0	0	0	0	1	0	1	1	1	0	1	1	0	0	0	0
0	1	1	0	1	0	0	1	1	0	0	0	0	1	1	1	1	1	1	0	1	0	1	1	1	1	1	0	0	0	0	0	0	1	1	1	1	0	1	1	1	0	1	1	1	1	0	1	1	1	0	0	1	0	0	1	1
0	1	1	0	0	0	0	1	1	0	0	0	0	1	1	1	1	1	1	0	1	0	0	0	1	1	1	0	1	0	0	0	0	0	0	1	0	0	1	1	1	0	1	1	1	1	1	1	1	1	0	0	1	1	0	1	0
1	0	1	0	0	0	0	1	0	0	0	0	0	0	1	1	1	1	1	0	0	1	0	0	1	1	0	1	1	0	1	0	0	1	1	1	0	0	0	1	1	0	1	0	0	1	1	0	1	1	1	0	1	0	0	0	0
1	0	1	0	0	0	1	1	0	1	0	0	0	0	1	1	1	1	1	0	0	1	0	0	1	1	0	1	0	1	1	1	0	1	1	1	0	0	1	1	1	0	1	0	0	1	0	1	1	1	1	0	1	1	0	0	0
1	1	1	0	0	0	0	1	0	1	0	0	0	0	1	1	1	1	1	0	0	0	0	0	1	1	0	1	0	1	1	0	1	1	1	1	0	0	1	1	1	0	0	0	0	1	0	1	1	1	1	0	1	0	0	1	0
0	1	0	0	1	1	1	1	0	1	0	0	0	1	1	1	1	1	0	1	0	1	0	0	1	1	0	1	0	1	1	1	0	1	1	1	0	1	0	1	1	0	0	0	0	0	1	1	1	1	0	1	1	1	0	1	0
0	1	1	0	1	1	1	0	0	1	0	0	0	1	1	1	1	1	0	1	0	1	0	0	1	1	1	1	0	1	1	0	1	1	0	1	0	1	1	1	1	0	0	0	0	1	0	1	1	1	1	0	1	1	0	1	0
0	1	0	0	1	1	1	1	0	0	0	0	0	1	1	1	1	1	0	0	0	0	0	0	1	1	1	1	0	1	1	0	0	0	0	1	0	0	1	1	1	0	1	0	0	1	0	1	1	1	0	1	1	1	0	0	0

ACOUSTIC DATA

Groups	DurPhra	Fmin	Fmax	Fdom	FDurSyl	FminSyl	FmaxSyl	DurSylTr	FdomTr	OrSyl	TotSyl
S. p. bryanti	1.7780	2590	6432	4583	0.1440	3759	4865	0.0970	4583	7	10
S. p. bryanti	2.1330	2673	7350	3632	0.1040	3007	4594	0.1420	4010	8	12
S. p. bryanti	1.5330	2423	8126	5188	0.0860	3341	5262	0.1150	5188	7	12
S. p. bryanti	1.5560	2339	8186	5102	0.1110	3341	5095	0.1060	5730	8	12
S. p. bryanti	1.5200	2423	8102	3286	0.1160	3425	5346	0.1030	4151	12	15
S. p. bryanti	1.8760	2757	7601	3545	0.1130	3843	5680	0.1260	3545	7	10
S. p. bryanti	1.1130	2757	8269	4324	0.1330	4594	6682	0.1190	4324	5	8
S. p. bryanti	1.1420	2924	8436	5534	0.1300	5534	7518	0.1450	5534	5	8
S. p. bryanti	1.6020	2673	7685	3718	0.1040	3007	6014	0.1450	5534	7	8
S. p. bryanti	2.0110	2339	7852	5707	0.1100	3007	4039	0.2220	3632	9	12
S. p. bryanti	2.1070	2506	8186	5880	0.1340	3091	4260	0.1930	4496	7	13
S. p. bryanti	2.0040	2339	8019	4410	0.1050	3174	4177	0.2140	3978	9	13
S. p. bryanti	1.5560	2339	8520	3459	0.1010	3675	5513	0.1580	3459	5	12
S. p. bryanti	1.6410	2506	6766	3891	0.0990	3592	4928	0.1160	6139	5	12
S. p. bryanti	1.6410	2357	6613	3891	0.1010	3675	4761	0.1180	6139	9	11
S. p. bryanti	1.4590	2757	8269	3891	0.1080	3843	6515	0.1950	3891	7	10
S. p. bryanti	1.5950	2005	8353	3891	0.1240	2840	5680	0.1070	3891	6	10
S. p. bryanti	2.0800	2506	8186	3718	0.0920	2840	5012	0.1540	4410	10	15
S. p. bryanti	2.1120	2256	8269	3372	0.1010	2506	4678	0.1720	3372	6	15
S. p. bryanti	2.0460	2339	8854	3286	0.1040	2506	4761	0.1620	3592	8	14
S. p. rufivertex	1.5250	2840	13364	3978	0.1090	3843	5513	0.1320	4842	6	10
S. p. rufivertex	1.3180	2339	17958	4496	0.1630	2924	9606	0.1410	5361	4	10
S. p. rufivertex	1.5020	2673	17874	3797	0.1190	3091	5095	0.1290	5448	6	11
S. p. rufivertex	1.5810	3007	19879	5015	0.1000	5262	7685	0.0950	5534	4	11
S. p. rufivertex	1.6890	3091	17123	6139	0.1040	5429	8019	0.0880	6139	5	11
S. p. rufivertex	1.3640	2840	14366	5621	0.1590	4427	6599	0.0950	5621	7	13
S. p. rufivertex	1.6730	2840	13197	5361	0.1670	4427	6515	0.0870	5361	7	11
S. p. rufivertex	1.2320	2590	14332	4842	0.1500	4427	6515	0.1460	4842	5	9
S. p. rufivertex	1.6050	2757	14700	5725	0.2000	4260	7518	0.1050	5275	7	12
S. p. rufivertex	1.6230	2673	14868	5621	0.0970	3174	5513	0.0810	5621	5	10
S. p. rufivertex	1.6940	3007	14116	5361	0.1690	4260	6348	0.0970	5361	8	12
S. p. rufivertex	1.6050	2757	15452	4842	0.1520	4427	6515	0.1350	4842	6	10
S. p. rufivertex	1.6660	3091	14366	6053	0.1630	3843	7267	0.1680	5361	4	9
S. p. rufivertex	1.5180	3007	12445	3978	0.1040	3592	6181	0.1410	5534	7	10
S. p. rufivertex	1.3870	3341	13531	4842	0.1860	3759	6682	0.1390	4064	6	11
S. p. rufivertex	1.2640	2506	14283	5015	0.1730	3675	6265	0.1290	5015	7	10
S. p. rufivertex	1.5280	2840	16538	5015	0.1440	5095	8269	0.0950	5534	6	10
S. p. rufivertex	1.5720	3258	12612	5534	0.1280	4761	5931	0.1520	5534	6	9
S. p. rufivertex	1.4530	2506	14533	4583	0.1470	3508	8520	0.1250	4842	9	12
S. p. rufivertex	1.4620	2757	17123	5361	0.1740	3341	7685	0.1190	3978	11	14
Island mangrove	1.5040	2590	16872	4237	0.0980	4260	4928	0.1230	4237	8	11
Island mangrove	1.2460	2673	16789	5188	0.1100	4260	5012	0.1360	5188	7	8
Island mangrove	1.4030	2590	17123	4151	0.1240	3759	5179	0.0890	5275	6	9
Island mangrove	1.9470	2924	17290	4410	0.0930	4594	7100	0.1180	4410	3	7
Island mangrove	1.6430	3341	18960	4669	0.0970	3759	5597	0.1240	4669	11	14
Island mangrove	1.5790	3091	19461	4756	0.0980	3258	5764	0.1400	4756	7	10
Island mangrove	1.5560	2840	15452	4064	0.1090	4678	7601	0.1410	4064	8	11
Island mangrove	1.2190	2840	18543	4929	0.1020	4678	5931	0.0860	4929	6	10
Island mangrove	1.9600	2757	18292	4496	0.1170	4928	6432	0.1230	4583	5	10
Island mangrove	1.6550	2590	18087	4583	0.0870	3592	5346	0.1330	4583	6	10
Island mangrove	1.4090	2590	19044	4669	0.1100	4511	8603	0.1340	4583	4	9
Island mangrove	1.6190	2673	17884	5275	0.1040	5429	7100	0.1460	5275	7	10
Island mangrove	1.9510	2840	16371	4064	0.1250	3843	6265	0.1140	4496	8	14
Island mangrove	1.6370	2423	13364	3805	0.1490	4761	6515	0.1540	5880	5	9
Island mangrove	1.7160	3007	12612	5102	0.1530	4761	6265	0.1680	5102	5	8
Island mangrove	1.7160	3007	16287	4064	0.1350	4010	5262	0.0870	4151	8	14
Island mangrove	1.9500	2840	11944	4151	0.1350	3926	6181	0.0640	4478	8	14
Island mangrove	1.8890	2924	13197	4410	0.1360	3759	6432	0.1190	5188	10	14
Island mangrove	1.9380	3007	15786	4496	0.1430	3843	6265	0.1160	5188	12	14
Island mangrove	1.9680	2757	12779	3978	0.1490	3926	6265	0.1260	3978	10	14
